# Endometrial cancer PDX-derived organoids (PDXOs) and PDXs with FGFR2c isoform expression are sensitive to FGFR inhibition

**DOI:** 10.1038/s41698-023-00478-6

**Published:** 2023-12-08

**Authors:** Asmerom T. Sengal, Vanessa Bonazzi, Deborah Smith, Cristian P. Moiola, Rohan Lourie, Rebecca Rogers, Eva Colas, Antonio Gil-Moreno, Sophia Frentzas, Naven Chetty, Lewis Perrin, Pamela M. Pollock

**Affiliations:** 1 Endometrial Cancer Laboratory, School of Biomedical Sciences, Faculty of Health, the Queensland University of Technology located at the Translational Research Institute (TRI), PA Hospital Campus, 37 Kent St Woolloongabba, Brisbane, 4102 QLD Australia; 2 Frazer Institute, The University of Queensland, Brisbane, QLD 4102 Australia; 3 Mater Pathology, Mater Health, Brisbane, QLD Australia; 4 Biomedical Research Group in Gynaecology, Vall Hebron Institute of Research (VHIR), CIBERONC, 08035 Barcelona, Spain; 5 Department of Medical Oncology, Monash Health, Melbourne, VIC Australia; 6 Faculty of Medicine, Nursing and Health Sciences and School of Clinical Sciences, Monash University, Melbourne, VIC Australia; 7 Department of Gynaecologic Oncology, Mater Cancer Centre, Mater Health Services, Brisbane, QLD Australia

**Keywords:** Endometrial cancer, Cancer models, Predictive markers, Translational research, Immunohistochemistry

## Abstract

Endometrial cancer (EC) patients with metastatic/recurrent disease have limited treatment options and poor survival outcomes. Recently, we discovered the FGFR2c splice isoform is associated with poor prognosis in EC patients. Here we report the establishment of 16 EC patient-derived xenografts (PDX)-derived organoids (PDXOs) with or without FGFR2c expression. In vitro treatment of 5 EC PDXOs with BGJ398 showed significant cell death in 3 models with FGFR2c expression. PDXs with high/moderate FGFR2c expression showed significant tumour growth inhibition (TGI) following 21-day treatment with FGFR inhibitors (BGJ398 or pemigatinib) and significantly prolonged survival in 4/5 models. Pemigatinib + cisplatin combination therapy (*n* = 5) resulted in significant TGI and prolonged survival in one of two p53abn PDXs. All five models treated with cisplatin alone showed de novo resistance and no survival benefit. Seven-day treatment with BGJ398 revealed a significant reduction in angiogenesis and CD206 + M2 macrophages. These data collectively support the evaluation of FGFR inhibitors in a clinical trial.

## Introduction

Endometrial cancer (EC) is the single gynaecological cancer that consistently shows a notable annual increase in both incidence and mortality in developed countries^1^. EC includes several histologic and molecular subtypes that have diverse prognostic outcomes. Traditionally, EC was classified as type I (well to moderately differentiated, endometrioid in histology associated with good prognosis) and Type II (poorly differentiated, non-oestrogen dependent with poor prognosis). In 2013, the Cancer Genome Atlas (TCGA) consortium identified four molecular subtypes with distinct prognostic significance^2^. These subtypes were subsequently validated using surrogate immunohistochemistry (IHC) biomarkers with Polymerase Ɛ-enzyme (*POLE)* hotspot mutation analyses. The McAlpine laboratory established the Proactive Molecular Risk Classifier for Endometrial Cancer (ProMisE), simplifying the TCGA approach^3^. The PORTEC consortium studies concurred with the ProMisE classification, except for minor variations in initial nomenclature^4^. All published studies concluded that patients with *POLE* exodomain mutant have excellent prognoses; mismatch repair deficient (MMRd) and p53 wildtype (p53wt)/no specific molecular profile (NSMP) have intermediate prognoses, and p53 abnormal (p53abn) have the worst prognoses^2,3,5^. The molecular classification is now endorsed by the World Health Organisation and several clinical trials are currently evaluating using molecular subtyping to tailor adjuvant treatment for EC patients^6^.

Women diagnosed with metastasis or recurrent disease have limited treatment options with <20% 5-year expected survival. The PORTEC-3 phase III clinical trial tested the addition of chemotherapy to radiotherapy compared to radiotherapy alone in high-risk women with EC^7^. Molecular subtyping of PORTEC-3 cohort tumour samples showed the combination of chemotherapy and radiotherapy increased survival for EC patients with the p53abn subtype but did not show significant benefit for patients with MMRd and NSMP/p53wt molecular subtypes^8^. The MMRd and p53wt/NSMP subtypes encompass 80% of diagnosed ECs contributing to 50% of EC deaths and therefore, need additional treatment optimisation. Recently, immune checkpoint inhibitors (ICIs) monotherapy, including pembrolizumab and durvalumab have shown efficacy in patients with MMRd ECs and are approved by regulatory bodies. The combination of pembrolizumab (anti-PD-1 antibody) + lenvatinib has been shown to increase survival by 6 months in 40% and 30% of MMRd and MMR proficient (MMRp) patients, respectively in a large phase III clinical trial^9^. Following this study, the combination regimen was granted accelerated approval in several countries, including the USA, Europe and Australia. However, the combination of pembrolizumab + lenvatinib is not biomarker-driven and 90% of patients developed significant treatment-related adverse effects^9^. Although lenvatinib has been reported to be a multitarget inhibitor with activity against Vascular Endothelial Growth Factor Receptors (VEGFRs) and Fibroblast Growth Factor Receptors (FGFRs), evidence for its multi-target effects has come from murine studies using 3–10 mg/kg dosing, which produces plasma concentrations that are 9–30 fold higher than those achievable in patients^10^.

FGFR2 is a member of the FGFR family and has two main isoforms, FGFR2b and FGFR2c which are expressed in normal epithelial and mesenchymal cells respectively^11,12^. FGFR2 can be dysregulated via mutation, amplification, or gene fusion in different solid cancers. We have identified *FGFR2* mutations in about 10–15% of EC and shown that mutations were associated with shorter progression-free survival (PFS) and disease-specific survival (DSS)^13^. In studies where molecular subtyping have been performed, FGFR2 mutations occur primarily in the MMRd and p53wt subtypes^2,5^. Recently, we discovered *FGFR2c* splice isoform expression due to isoform switching in ~50%, ~30% and ~20% of patient samples with MMRd, p53wt and p53abn molecular subtypes of EC, respectively^14^. We demonstrated *FGFR2c* was an independent prognostic biomarker associated with shorter PFS and DSS compared to FGFR2b expressing tumours in the MMRd and p53wt subtypes but not in the p53abn and *POLE* mut subtypes^12^. FGFR2c signalling contributes to epithelial-to-mesenchymal transition (EMT), enhances cell motility and invasiveness and inhibits tumour differentiation in several cell lines^15–18^.

Previously, our laboratory demonstrated EC cell lines harbouring FGFR2 mutations are oncogene addicted and are sensitive to FGFR inhibition (PD173074 and BGJ398) in vitro^19,20^ and in vivo using cell line xenograft models^20,21^. Recently, the US Food and Drug Administration (FDA) has approved infigratinib (BGJ398) and pemigatinib in intrahepatic cholangiocarcinoma with FGFR2 fusions, as well as erdafitinib in advanced urothelial cancer with FGFR2/3 alterations^22,23^. Although we have identified FGFR2c isoform switching as a new mechanism of receptor activation in EC that is associated with poor prognosis, there is no functional data showing ECs with FGFR2c isoform expression show oncogene dependence and the role of FGFR inhibitors in preclinical models with FGFR2c expression is unknown.

Despite the advances in molecular profiling of EC, the discovery of effective novel targeted therapies for endometrial cancer is lagging, partly due to a lack of robust preclinical models that reflect the spectrum of molecular subtypes. Patient-Derived Xenografts (PDXs), PDX-derived organoids (PDXOs) and Patient-Derived Organoids (PDOs) are robust models for preclinical drug testing, that better mimic patient tumour heterogeneity and molecular profiles.

The objectives of this investigation were: 1) to establish PDXOs from multiple EC PDX models representing advanced ECs; 2) to characterise the established PDXs and PDXOs and identify appropriate models with and without *FGFR2c* expression that can be targeted with FGFR inhibitors; 3) to assess the in vitro and in vivo efficacy of FGFR inhibitors and 4) to test if FGFR inhibition sensitises to cisplatin, in *FGFR2c* expressing EC PDXs representing various histologic and molecular subtypes.

## Results

### Characterization of EC PDXs and PDXOs

**Fig. 1:**
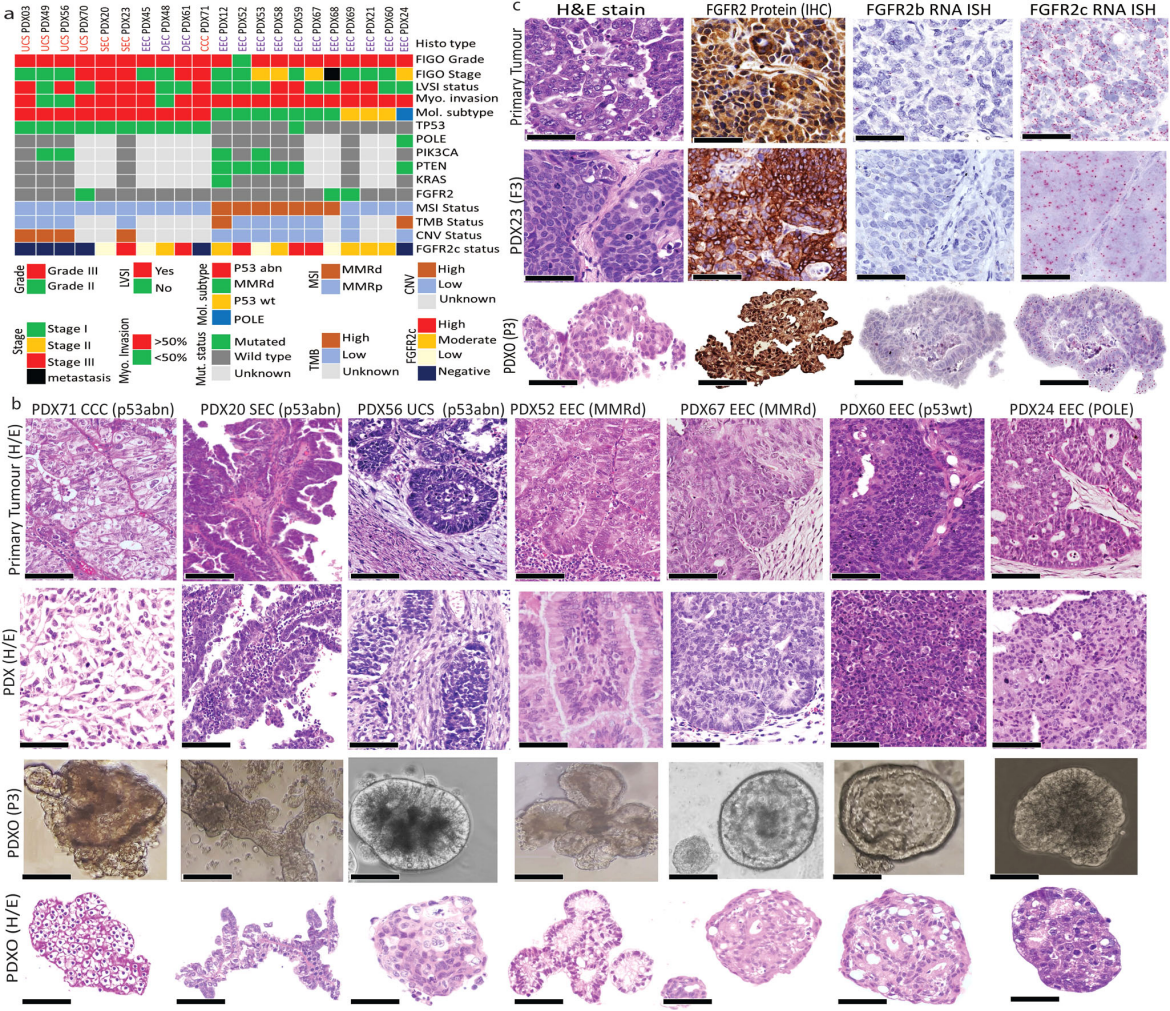
Characterization of patients’ primary endometrial cancer, PDXs and PDX-derived organoids (PDXOs). **a** Histologic and molecular characterization of 21 endometrial cancer PDXs **b** Representative images from different histologic and molecular subtypes of endometrial cancer showing 7 primary patients’ tumour with different histologic and molecular types (top panel) recapitulated their morphology in corresponding PDXs (upper middle panel) and PDXOs (light microscope captured micrography of individual PDXOs, lower middle panel) and (H/E stained morphology of PDXOs, bottom panel). **c** Representative images for PDX23 demonstrating tumour histologic morphology (H/E), FGFR2 protein expression detected via IHC with a pan-FGFR2 antibody and FGFR2b and FGFR2c mRNA expression in patients’ primary tumour (upper panel), PDX 23 tumour passage 3 (F3) (middle panel) and corresponding PDXO23 at passage 3 (P3) (lower panel); Scale bar indicates 50 µm. EEC endometrioid endometrial carcinoma, DEC dedifferentiated endometrial carcinoma, CC Clear cell carcinoma, UCS uterine carcinosarcoma, CNV Copy number variation, FIGO International Federation Gynaecological Oncology, FGFR2c Fibroblast growth factor receptor 2c splice isoform, MSI microsatellite instability, MMRd mismatch repair deficient, LVSI Lymphovascular space invasion, Mol Molecular, Myo, Myometrial, TMB Tumour mutation burden, PDX patient-derived xenograft, PDXO patient-derived xenograft organoid, IHC Immunohistochemistry, H/E haematoxylin/eosin.

We have previously published detailed genomic profiling for 11 EC PDXs alongside the establishment of another 7 PDX models^24^. In the current study, we report RNA-ISH and IHC characterisation of 21 PDXs, including 3 PDXs originally established at Vall Hebron Institute of Research (VHIR), Spain. As PDXOs are more scalable and cost-effective platforms than PDX models for evaluating new drugs and drug combinations, we sought to generate PDXOs from our histologically and genomically characterised PDXs. The clinicopathologic characteristics and follow-up with clinical outcomes of patients who donated their tumours for establishment of these PDXs and matched PDXOs are provided in Supplementary Table 1. The sketch for evaluation of morphological, IHC and genomic characterisation and biobanking of the models are outlined in Supplementary Fig. 1. The cohort of PDX and PDXOs represent the various histologic subtypes, including the rare aggressive histologic types of EC with limited preclinical models and the four ProMiSE molecular subtypes of EC (Fig. 1a).

We successfully generated and biobanked PDXOs from 16/17 (94 %) of our PDX models. The established PDXOs also capture the various histologic subtypes of EC, including endometrioid EC (*n* = 10), serous EC (*n* = 2), dedifferentiated ECs (*n* = 1), clear cell (CC) carcinoma (*n* = 1) and uterine carcinosarcoma (UCS) (*n* = 2). The cohort of PDXOs also represent all molecular subtypes, including p53abn (*n* = 7), MMRd (*n* = 6), p53wt (*n* = 2), and *POLE* mut (*n* = 1). Nearly all the established PDXOs recapitulated the morphological and molecular patterns of the corresponding patients’ primary tumours and PDXs (Fig. 1b). UCS models (PDXO49 and PDXO56) retained the carcinomatous and sarcomatous components that were observed in the original patient tumours and PDXs (Supplementary Fig. 2). The sarcomatous features were more evident following the removal of the ROCK inhibitor and the addition of 5 µg/ml heparin sulphate (HS) and 50 µg/ml ascorbic acid to the organoid media to support the growth of the sarcomatous component^25^ (Supplementary Fig. 2). For those models with available patient tumour sections, the corresponding PDXs and PDXOs samples were assessed for oestrogen receptor (ER) and progesterone receptor (PR) expression. While most of the tested models had very low hormone receptor expression (<1%), high to moderate ER expression was observed in only 3 models (Supplementary Fig. 3) and 2/3 ER+ models were FGFR2c negative indicating that most of our PDX/PDXO models represent hormone-independent aggressive ECs.

**Fig. 2:**
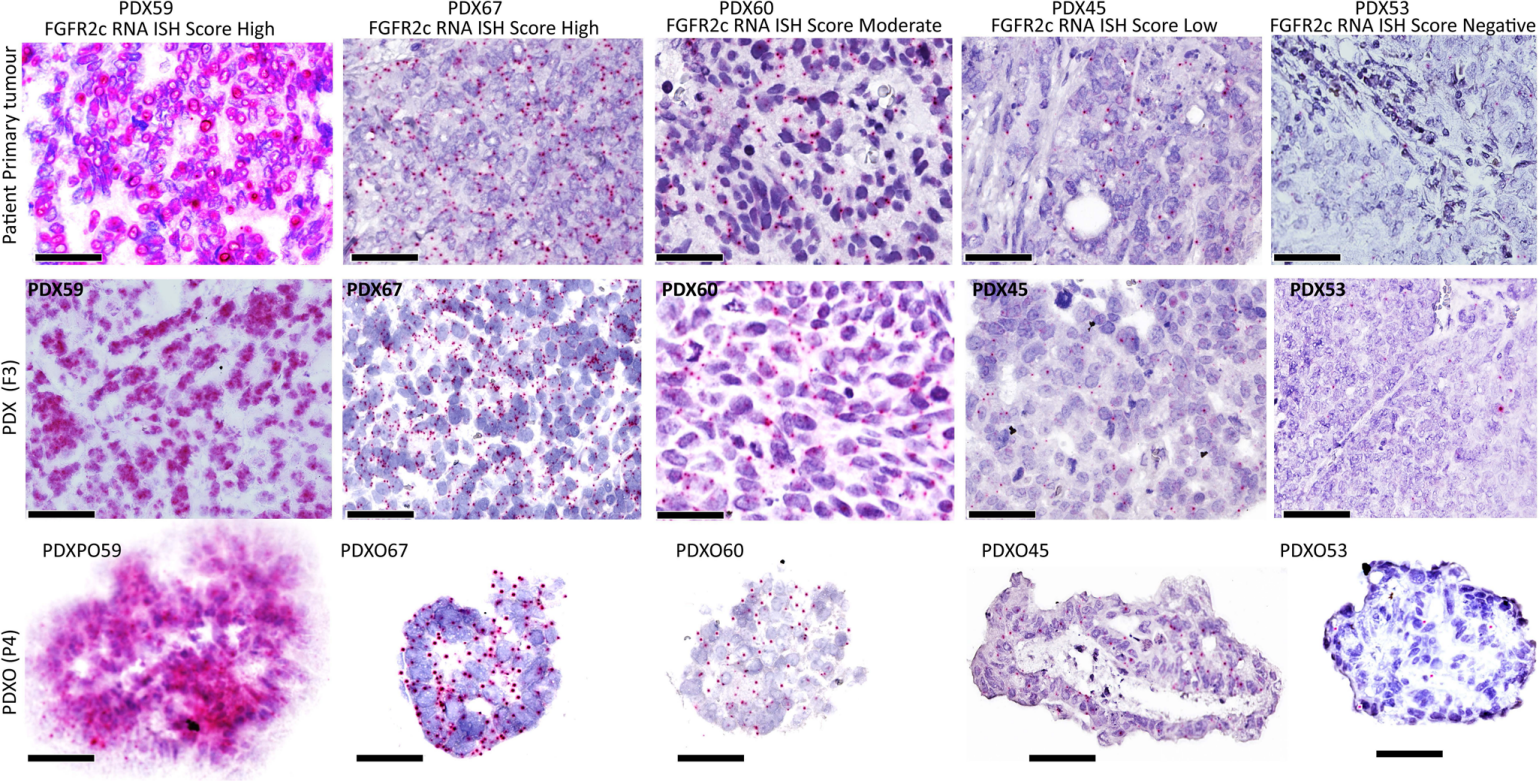
Different levels of expression of FGFR2c revealed by RNA ISH showing FGFR2c expression in PDXs and PDXOs recapitulate corresponding patient tumours. The micrograph images illustrate five EC patients and corresponding PDXs and PDXOs representing the different patterns of FGFR2c splice isoform expression. The first column represents the single model with very high FGFR2c splice isoform expression with an RNA ISH signal score of 4+ (cluster of signals without distinct dots or >10 clusters). The second column represents models with high FGFR2c expression (RNA ISH score of 3+ (>10 signals/cell with ≤10 clusters). The third column represents a model of moderate FGFR2c expression with an RNA ISH score of 2+ (4-10 signals/cell without clustered dots) the fourth column represents a model with a low FGFR2c splice isoform RNA ISH score of 1 (2-3 signals/cell or >1 signals/10 tumour cells) and the fifth column is a model with negative FGFR2c splice isoform with RNA ISH score of 0 (<1 signal/10 tumour cells).

FGFR2 isoform status was determined for each primary patient tumour and matched PDX and PDXO via our published BaseScope RNA ISH assays which detect the FGFR2b and FGFR2c splice isoforms^14^. The expression of the *FGFR2c* splice isoform was highly consistent between the primary patient tumour and the matched PDXs and PDXOs (Figs. 1c and 2). We observed four patterns of *FGFR2c* expression high (*n* = 5), moderate (*n* = 6), low (*n* = 4) and negative (*n* = 6) (Fig. 1a) and representative images of patient primary tumours and matched PDXs and PDXOs showing these patterns are provided in Fig. 2. There was no significant difference in morphology and proliferation across multiple passages of the PDXOs (P3-P10) and multiple rounds of freeze-thawing cycles did not impact the organoid growth pattern, viability, and morphology (Supplementary Fig. 4). For 8 PDX models, PDXOs were generated from 3 or more independent mice carrying PDX tumours at multiple passages (F3-5) to ensure some in vitro functional experiments and drug testing could be performed in biological triplicate.

### Differential ligand dependence of EC PDXOs

**Fig. 3:**
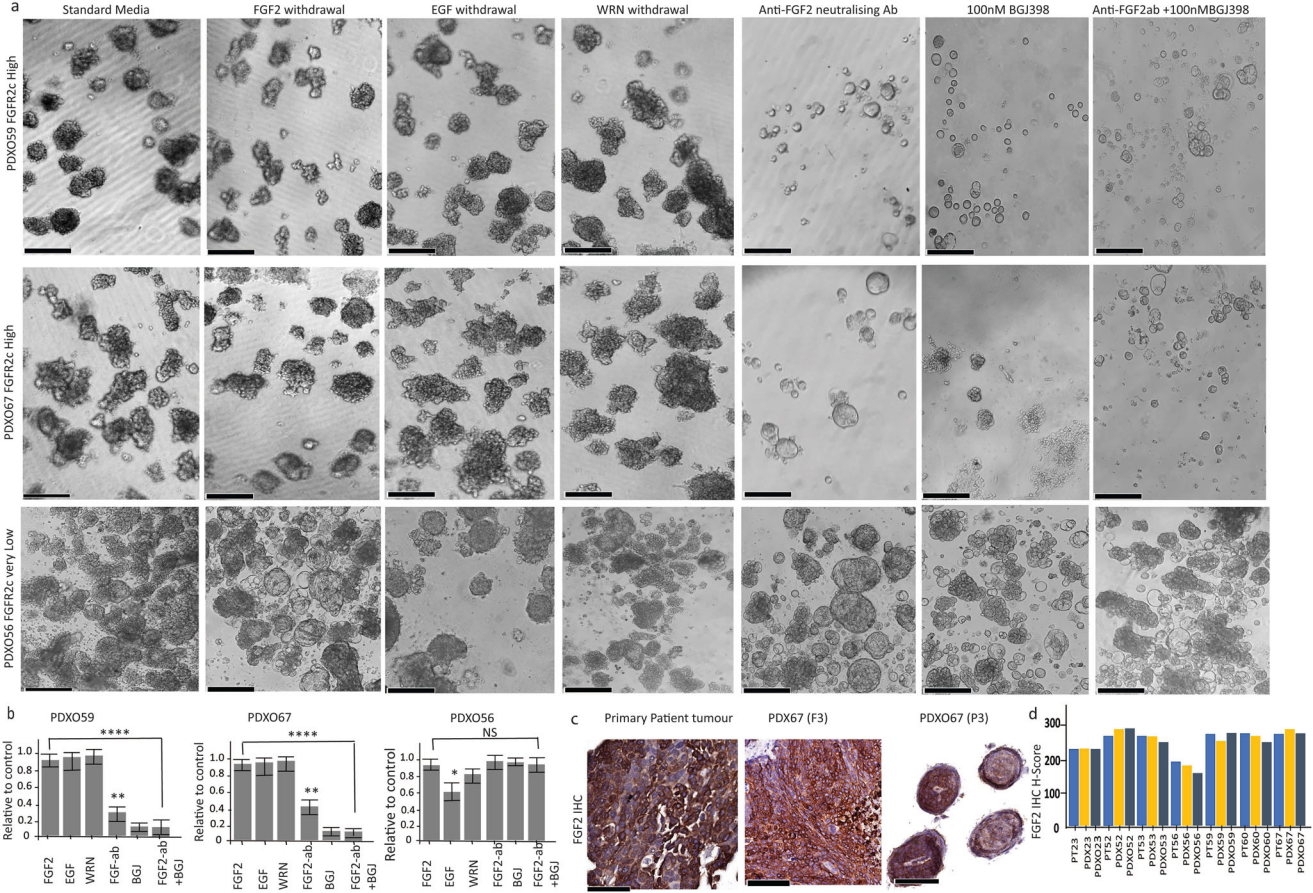
Regeneration and growth of EC PDXOs expressing FGFR2c are independent of exogenous growth factors but dependent on endogenous FGF2. **a** Representative images of EC PDXO59 (upper panel), PDXO67 (middle panel) and PDXO56 (lower panels) cultured for 14 days in standard organoid media with the indicated withdrawal of growth factors and treatment with neutralising anti-FGF2 antibody alone or in combination with FGFR inhibitor (BGJ398). **b** Quantitation of relative growth reduction in three PDXOs cultured with the indicated withdrawal of growth factors or treatment with anti-FGF2 neutralising antibody, BGJ398 and both anti-FGF2 neutralising antibody and BGJ398. This experiment was performed in independent biological triplicate using independent PDXOs established from three different mice carrying each PDX. Organoid counting was performed by capturing 3 independent fields at a 10X microscopic objective view of each experiment. The error bar indicates the standard error of the mean (SEM) of the biological triplicates. Significance was assessed using a one-way ANOVA with Dunnett’s multiple comparison test. * *P* < 0.01, ** *P* < 0.001, **** *P* < 0.00001. **c** Representative IHC images showing expression of FGF2 in primary tumours and corresponding PDX and PDXO. **d** FGF2 IHC H-Score of primary patient tumour and matched PDXs and PDXOs. The scale bar indicates 200 µm for the PDXOs and 50 µm for the IHC. Ab antibody, PDX Patient-Derived Xenograft.

Generic stem cell organoid media contains a variety of growth factors and supplements to facilitate organoid proliferation and regeneration, including EGF, FGF2, as well as WNT3A, R-Spondin and Noggin (WRN). Our standard organoid media did not contain several of the supplements included in previous reports of PDOs generated from normal endometrial epithelia and ECs^26–28^. Based on our hypothesis that the splicing switch to FGFR2c establishes an autocrine loop in EC cells, we performed growth factors (GFs) withdrawal experiments in PDXO59 and PDXO67 with FGFR2c expression and PDXO56 without FGFR2c expression (Fig. 3a, b). Withdrawal of indicated GFs 24 h following seeding showed that EC PDXOs with high FGFR2c expression (PDXO59 and PDXO67) did not require exogenous FGF2, EGF or WRN however, they were susceptible to treatment with 25 µg/ml anti-FGF2 mAb and/or 100 nM BGJ398 and significant growth reduction and morphologic changes were evident (Fig. 3a upper and middle panels and b). All PDXOs with FGFR2c expression were subsequently cultured without the addition of exogeneous GFs, including FGF2, EGF and WRN and the growth pattern was not altered. In contrast, PDXO56 without FGFR2c expression showed a significant reduction in proliferation following removal of EGF, however, treatment with anti-FGF2 antibody and/or BGJ398 had no impact (Fig. 3a lower panel, and b). Although PDXO56 represents a UCS with potentially different disease aetiology, PDXO53 from an endometrioid EC with very low FGFR2c expression was similarly resistant to BGJ398 (see results below). The neutralising FGF2 antibody data suggested that the initial growth of EC PDXOs with FGFR2c expression was dependent on endogenous FGF2 via the establishment of an autocrine loop (Fig. 3a, b). Indeed, IHC analyses of FGF2 on 7 primary patient tumours and corresponding PDXs and PDXOs revealed high FGF2 expression, independent of their FGFR2c status (Fig. 3c, d). To confirm the specificity, the FGF2 antibody was validated via western blot analyses in BaF3 cell lines stably transduced with a subset of individual FGF ligands (Supplementary Fig. 5b).

**Fig. 4:**
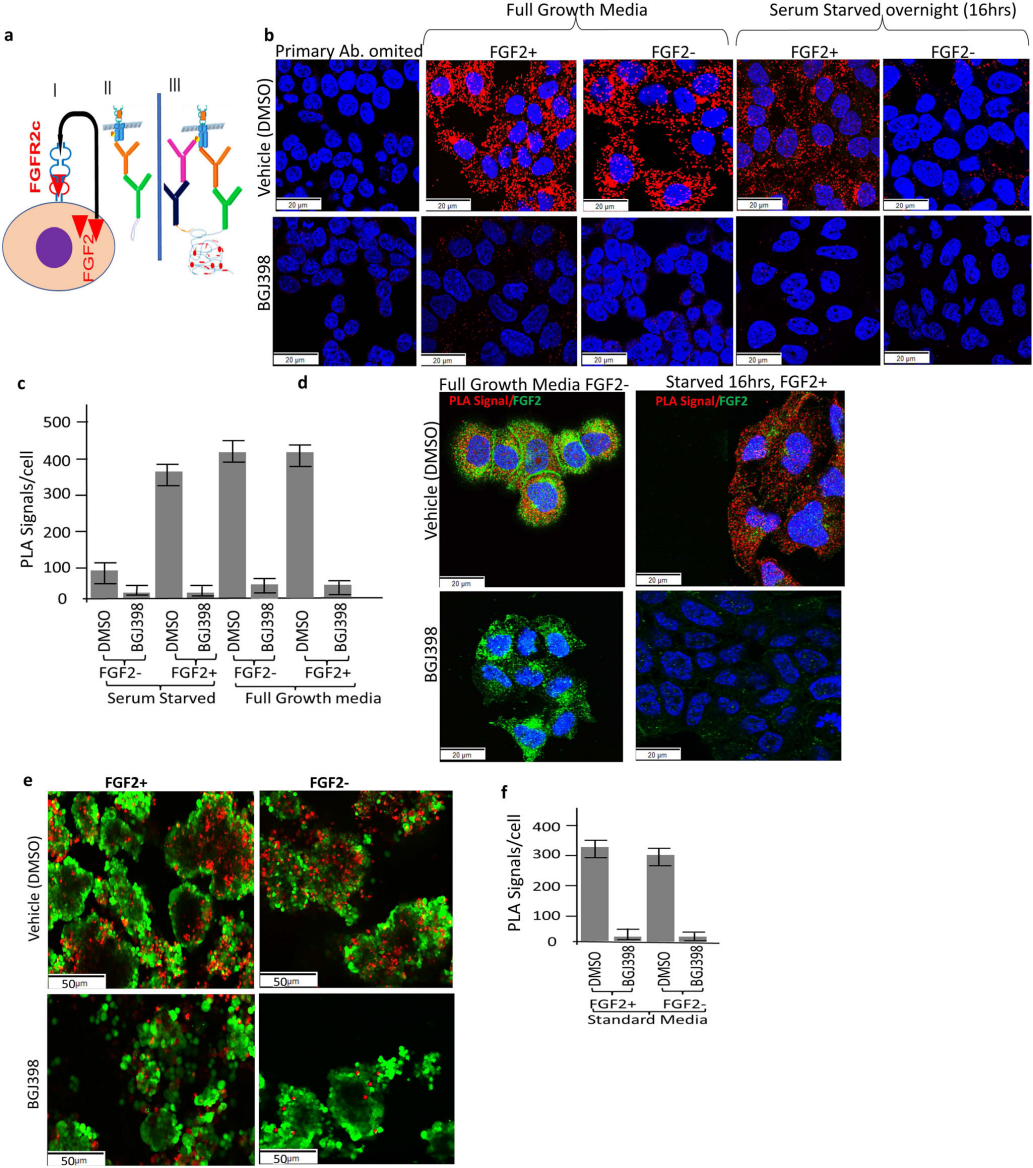
In situ detection of phosphorylated FGFR2c using PLA to demonstrate FGF2/FGFR2c autocrine loop signal activation in PDXO-derived cell line and PDXO. **a** Schematic diagram illustrating the principle of PLA assay and autocrine loop mode of activation in carcinoma cells with FGFR2c splice isoform expression (I); In situ indirect PLA assay design, with one primary antibody omitted (technical negative control) without signal formation (II) and positive signal formation with incubation of both primary antibodies (III). **b** Representative images of PLA assay in ASPDX67_CL primary EC cell line grown in DMEM/F12 with 10% FBS or 16 hr of serum starvation (0.5% FBS) followed by stimulation with and without 10 ng/ml FGF2 + 0.01% DMSO for 30 min (top panels) or 300 nM BGJ398 (bottom panels). **c** Representative images demonstrating co-expression of FGF2 and pFGFR2c in the vehicle and BGJ398 treated ASPDX67_CL primary EC cell line. FGF2 is markedly reduced when cells were serum starved (left panels). **d** Reduction in pFGFR2c PLA signal/cell in ASPDX67_CL primary endometrial cancer cell line in different conditions. **e** Representative images of PLA results in PDXO67 cultured in standard organoid media without GFs +/- 10 ng/ml FGF2 and either vehicle-treatment (left panels) or BGJ398-treatment (right panels). **f** Quantitation of pFGFR2c signal in EC PDXO67 with or without FGF2 in the vehicle and BGJ398 treated organoids. All PLA signal data analyses were performed using automated Fiji ImageJ2. Error bars indicate the standard error of the mean (SEM) from technical triplicates. Small red dots indicate PLA signals of pFGFR2c and blue indicates nuclei stained with DAPI. Green IF in the PDXOs shows Histone-3 nuclear stain. EC endometrial cancer; FGF2, Fibroblast Growth Factor; PLA, proximity ligation assay; pFGFR2c, phosphorylated Fibroblast Growth Factor Receptor 2c splice isoform.

For PDX67, initial culturing of the PDXO in 2D culture without Matrigel led to the development of an adherent EC primary cell line, ASPDX67-CL (named with the first author initials and the PDX number). To confirm that FGFR2c was activated via endogenous FGF2 in EC, this cell line was incubated for 30 min with 5 µg Heparin Sulphate (HS) with and without 10 ng/ml FGF2 in the presence of vehicle (DMSO) or 300 nM BGJ398 in full growth media (10% FBS in DMEM/F12) or following 16 hr overnight serum starvation in 0.5% FBS and receptor phosphorylation was determined via the Proximity Ligation Assay (PLA) (Fig. 4). High PLA signals were observed in cells stimulated with HS + FGF2 in full-growth media however, similar signals of PLA were also noted without the addition of exogenous FGF2 (Fig. 4b, c). No PLA signals were seen when BGJ398 was added, confirming the specificity of the PLA for the detection of phosphorylated FGFR2c. Following serum starvation for 16 h, high PLA signals were seen with stimulation of exogenous FGF2 but not in the absence of FGF2, suggesting that serum starvation reduces FGF2 expression. The reduction in endogenous FGF2 expression following serum starvation was then confirmed by IF in this cell line model (Fig. 4d). We then performed PLA to confirm the autocrine activation of FGFR2c by endogenous FGF2 in the PDXO67 organoid. When organoids were cultured in standard organoid media without GFs + 10% FBS with and without the addition of exogenous FGF2 they showed almost equal number of PLA signals (Fig. 4e, f). To investigate further the signalling activation downstream of FGFR2c, we also examined the expression of pERK1/2 and pSTAT3 in BGJ398-treated ASPDX67-CL grown in 2D and PDXO67 cells grown in 3D using phospho-specific antibodies via in situ immunofluorescence (IF). Notably, BGJ398-treated ASPDX67-CL cells and PDXO67 demonstrated a significant reduction in pERK1/2 and pSTAT3 expression compared to vehicle-treated samples (Supplementary Fig. 6).

**Fig. 5:**
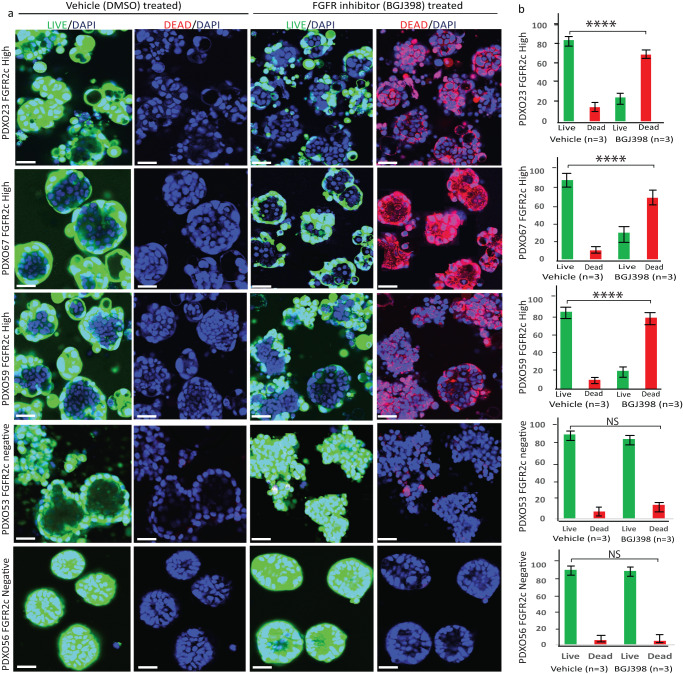
Significant cell death was observed in established PDXO models expressing FGFR2c following 72 hr treatment with 300 nM BGJ398. **a** Representative images from three different PDXO models expressing FGFR2c (top three panels) and two PDXO models without FGFR2c expression (bottom two panels) treated with vehicle (DMSO) or 300 nM BGJ398 for 72 h. Images were captured in XYZ stack at 10x microscopic objective view airy using an Inverted Laser rotatory Confocal Fluorescent Olympus Microscope with (FV1200 software). Green fluorescence shows esterase activity of live cells (Calcein AM), red fluorescence is generated upon binding of Ethidium homodimer-1 to DNA in damaged cells and nuclei were stained with DAPI. **b** Bar graph showing the proportion of live (green) and dead (red) cells in 5 independent PDO models treated with DMSO vehicle and 300 nM BGJ398. The experiment was performed in independent biological triplicate using independent PDXOs established from three different mice carrying each PDX as well as in technical triplicate. Live and Dead image analyses were performed using automated Fiji ImageJ2 software. **** two-sided student’s T-test *P* < 0.0001; Error bars indicate standard error of the mean (SEM), Scale bar 20 µm. DMSO Dimethyl-sulphoxide, DAPI Diamidino-2-phenylindole, FR2c + , Fibroblast Growth Factor Receptor 2c positive, FR2c- Fibroblast Growth Factor Receptor 2c negative, PDOs Patient-Derived Organoids, PDX Patient Derived Xenograft. NS not statistically significant.

While data in Fig. 3 showed initial culturing of organoids was sensitive to 100 nM BGJ398, this does not reflect the treatment of well-established cancers in patients. Therefore, we generated well-established organoids grown from PDXO67 (FGFR2c high) and PDX56 (FGFR2c negative) for 10 days and tested increasing concentrations of BGJ398 at different time points (24, 48 and 72 hr) prior to assessing viability, morphology and proliferation status. In this context, 100 nM BGJ398 had a limited effect on organoid morphology, proliferative capacity, and viability at any time point (Supplemental Fig. 7a–d). However, treatment with 300 nM BGJ398 for 72 hr revealed a marked reduction in proliferation with evident cell death/necrosis and a cystic morphology in PDXO67 (Supplemental Fig. 7a–d) which was not noted in PDXO56 (Supplemental Fig. 7c, d). Finally, five independent PDXO models were treated with 300 nM BGJ398 or 0.01% DMSO vehicle for 72 h. The three EC PDXOs with high FGFR2c isoform expression were established from PDX59 and PDX67 (MMRd) and PDX23 (p53abn) and the two PDXOs with low or negative FGFR2c isoform expression were established from PDX53 (EEC MMRd) and PDX56 (UCS p53abn). To ensure the most robust results, three independent organoid cultures were derived from three different mice each carrying the individual PDXs to represent true biological replicates. Using the LIVE/DEAD^TM^ assay followed by high-content automated image analyses, significant cell death (*P* < 0.0001) was observed in models expressing high FGFR2c isoform (PDXO23, PDXO67, PDXO59) but not in those with FGFR2c negative/low expression (PDXO56 and PDXO53) or vehicle-treated organoids (Fig. 5).

### Validation of *FGFR2c* expression as the molecular target via *FGFR2* shRNA mediated knockdown in EC PDXOs

**Fig. 6:**
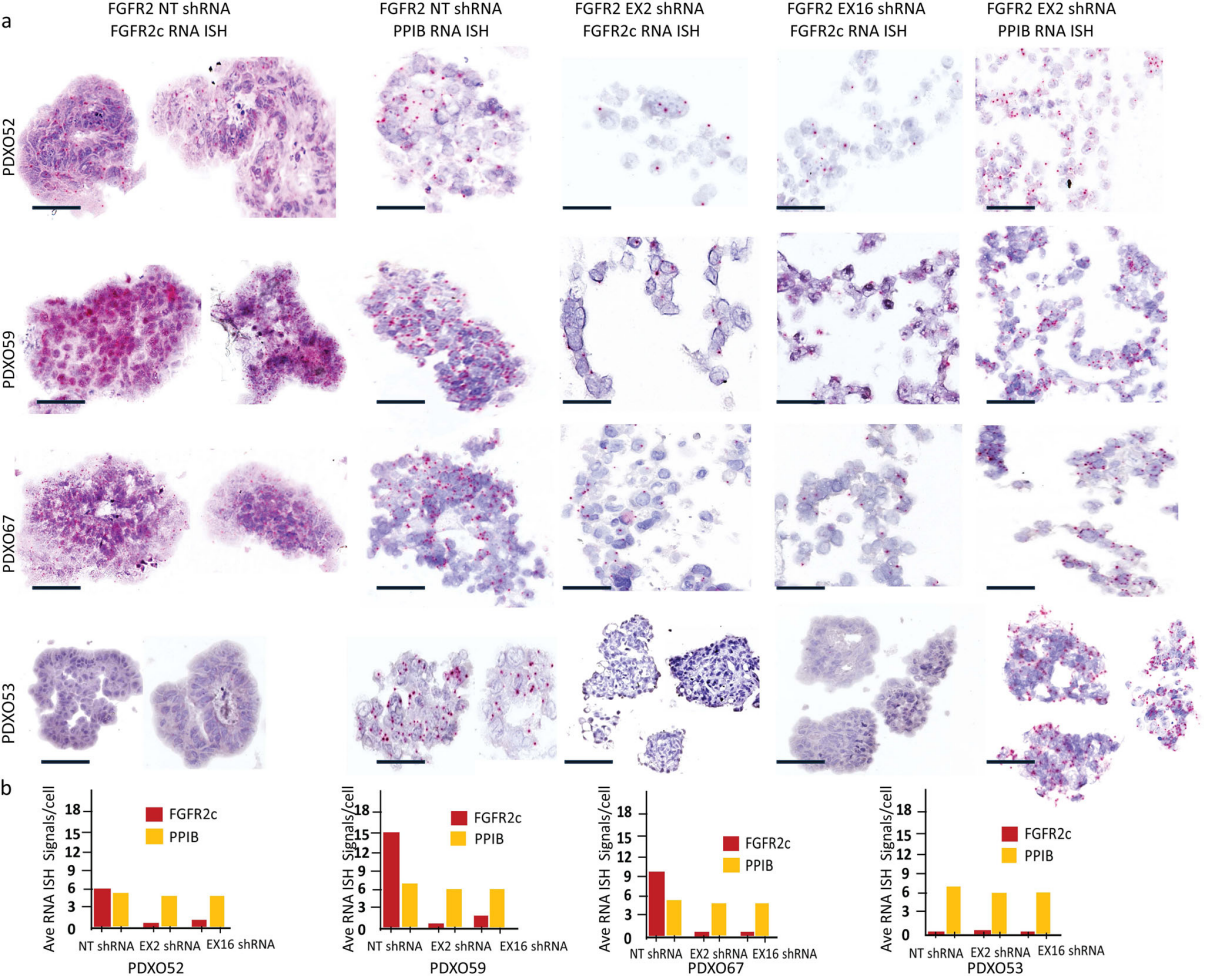
*FGFR2c* expression relative to Peptidylprolyl isomerase B (PPIB) a housekeeping gene following *FGFR2* shRNA mediated knockdown in four endometrial cancer PDXO models. **a** Representative micrography images of *FGFR2c* mRNA expression (first, third and fourth column) and *PPIB* a housekeeping gene (second and fifth column) detected via BaseScope RNA ISH on indicated endometrial PDXO models. **b** Analyses showing *FGFR2c* RNA ISH signals relative to *PPIB* (a housekeeping gene) in four endometrial cancer PDXOs. BaseScope RNA ISH was performed on FFPE organoid blocks collected 6 days after antibiotic selection. The scale bar indicates 50 µm. EX Exon, FFPE Formalin fixed paraffin embaded, FGFR2c Fibroblast growth factor receptor 2c splice isoform, NT nontargeting; PDXO Patient-derived xenograft organoids, PPIB Peptidylprolyl isomerase B.

**Fig. 7:**
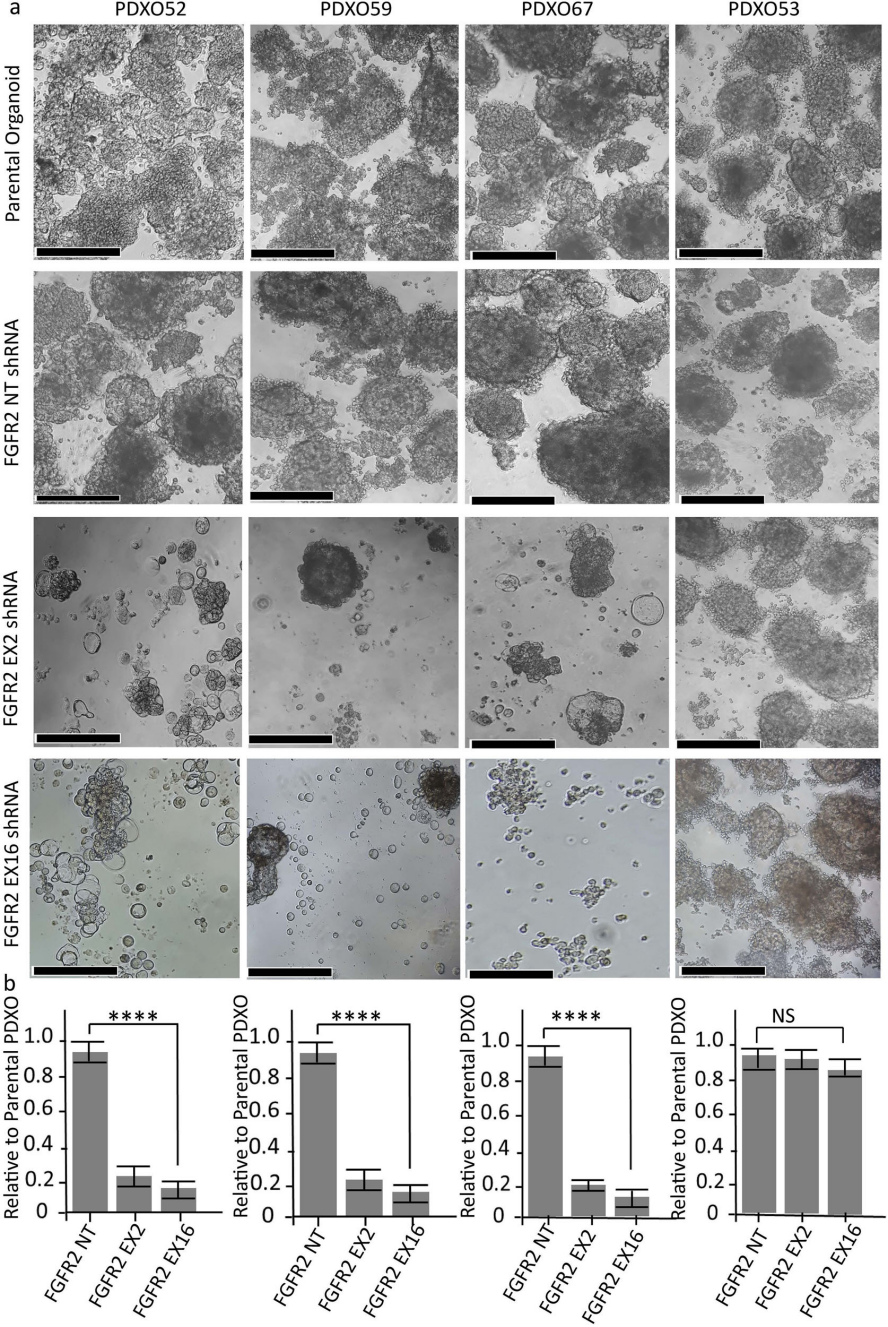
shRNA mediated *FGFR2* knockdown significantly reduces endometrial cancer PDXOs growth and viability. **a** Representative micrography images of three indicated EC PDXOs with high expression of *FGFR2c* mRNA and one without *FGFR2c* expression (biological control). Lentiviral transduction of PDXOs with two independent FGFR2 shRNA knockdown constructs targeting exon 2 and exon 16 showed a significant reduction in the growth of EC PDXOs (52, 59 and 67) which was not evident in PDXO53 (right panel). Untransduced PDXOs and those transduced with non-targeting (NT) shRNA have similar patterns of growth (upper 1^st^ and 2^nd^ panels) indicating lentiviral transduction alone has no off-target impact on the PDXO growth. **b** Quantitation of PDXO size and number 10 days following transduction of NT or FGFR2 shRNA relative to untransduced organoids for each PDXO model from 3 biologically independent experiments. Significant reduction **** *P* < 00001 (one-way ANOVA test) was noted in PDXO52, PDXO59 and PDXO67 with high *FGFR2c* expression in contrast to that seen in PDXO53 without *FGFR2c* expression. Viability was determined using Live/Dead assay and quantification was performed using ImageJ2. A scale bar indicates 100 µm, error bar indicates the standard error of the mean (SEM). EC endometrial cancer, EX exon, NS not significant, NT non-targeting, PDXO Patient-Derived Xenograft Organoids, sh short hairpin.

As BGJ398 inhibits FGFR1-3, we assessed the oncogenic dependency of these models on FGFR2c specifically, by knocking down *FGFR2* using two independent shRNA constructs (targeting exon 2 and exon 16 of *FGFR2*) in multiple EC PDXOs. Successful *FGFR2* knockdown was confirmed by determining the expression *FGFR2c* using BaseScope RNA ISH (Fig. 6a, b). *FGFR2c* was used as a surrogate of *FGFR2* as the PDXOs predominantly express *FGFR2c* splice isoform. *FGFR2* shRNA mediated knockdown resulted in a significant reduction in growth, regeneration, and viability in PDXOs with high FGFR2c expression (PDXO52, 59 and 67) but had no impact on PDXO53 without FGFR2c expression (Fig. 7a, b). PDXOs with *FGFR2* knockdown failed to propagate on subsequent passaging. FGFR1 and FGFR3 expression was determined via IHC on the primary patient tumour, PDXs and PDXOs and very low expression of FGFR1 and FGFR3 were noted in those with high FGFR2c expression (Supplementary Fig. 8a, b) confirming that the FGF2/FGFR2c autocrine loop is the target of BGJ398 in these models. Notably, PDXO56 showed relatively higher expression of FGFR1 and FGFR3, but no evident response to pan-FGFRi.

### In vivo targeting of EC PDXs with Infigratinib/BGJ398 and Pemigatinib FGFR inhibitors

**Fig. 8:**
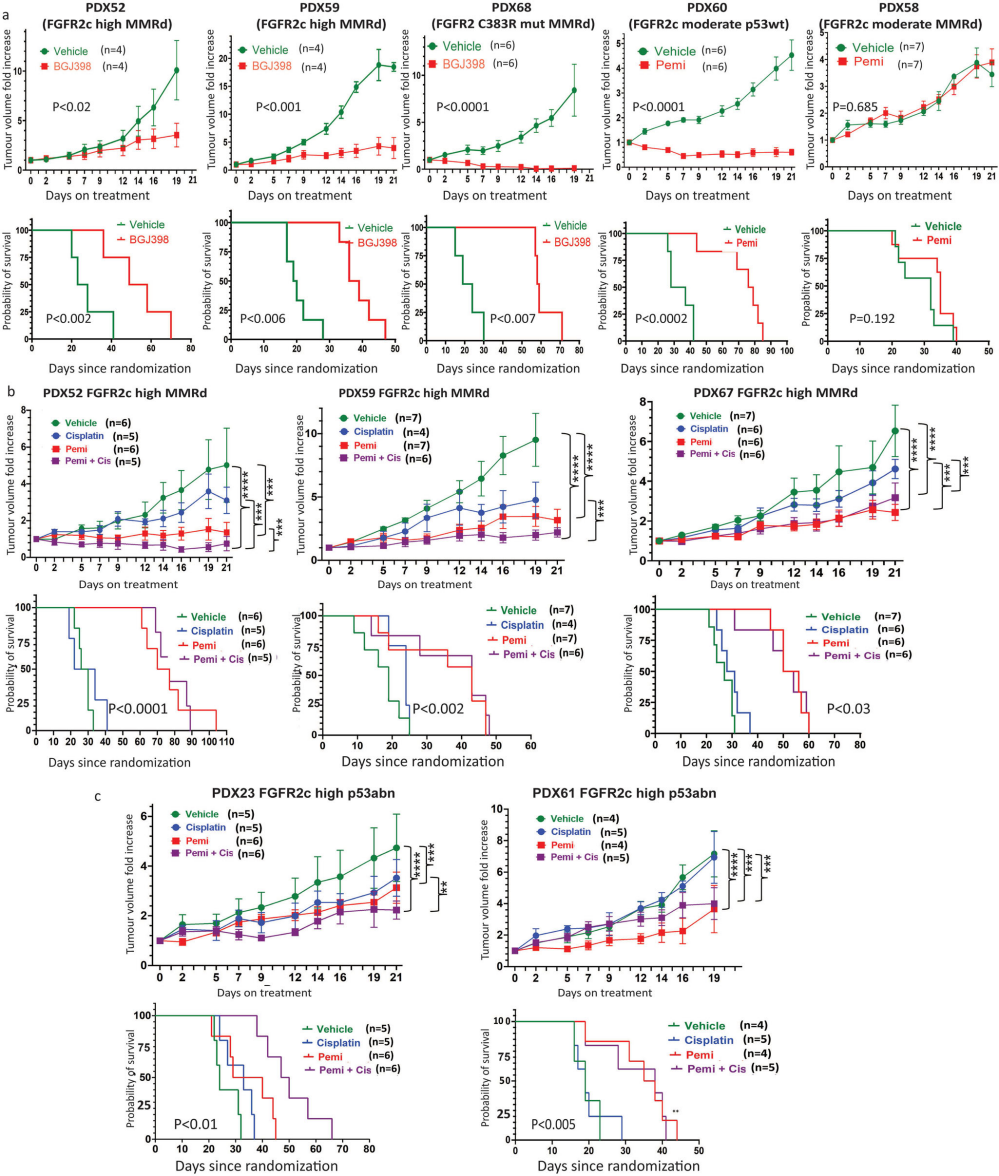
In vivo data showing significant tumour growth inhibition (TGI) and longer survival in EC PDXs with FGFR2 activation treated with FGFRi alone or in combination with Cisplatin. **a** Five independent EC PDX models treated with either BGJ398/infigratinib (PDX52, PDX59 and PDX68) or pemigatinib (PDX60 and PDX58). For each model, tumour growth inhibition (upper panel) and survival data (lower panel) are shown. **b** Three independent EC PDX models with FGFR2c expression representing the MMRd molecular subtype were treated with cisplatin alone, pemigatinib alone or pemigatinib plus cisplatin with tumour growth inhibition (upper panel) and survival data (lower panel) presented for each PDX model. **c** Two independent EC PDX models with FGFR2c expression and representing the p53abn molecular subtype were treated with cisplatin alone, pemigatinib alone or pemigatinib plus cisplatin and tumour growth inhibition (upper panel) and survival data (lower panel) are presented. Significance for TGI was assessed with a two-way ANOVA for models without any missing tumour measurements and a mixed model for those with one or more missing tumour measurements. A Tukey correction was performed for multiple comparisons and the Geisser Greenhouse correction was used for unequal variance. Significance for survival curves was assessed with a log-rank test * *P* < 0.05, ** *P* < 0.001 *** *P* < 0.0001, **** *P* < 0.00001. ANOVA, Analysis of variance; cis, cisplatin; EC, endometrial cancer, PDX, patient-derived xenograft.

Both infigratinib (BGJ398) and pemigatinib are orally bioavailable FGFR1-3 specific inhibitors that are approved as second-line treatment for patients with advanced intrahepatic cholangiocarcinoma with *FGFR2* dysregulation. To validate our findings in vivo, the matched PDXs from one in vitro sensitive model plus an additional 4 models were treated with either 30 mg/kg BGJ398 (PDX52 and PDX59, MMRd FGFR2c high and PDX68 MMRd, FGFR2 mutant) or with 1 mg/kg pemigatinib (PDX58 MMRd and PDX60 p53wt both with moderate FGFR2c expression) daily for 21 days. Four of the five PDX models treated with FGFRi showed significant tumour growth inhibition (TGI) and had significantly longer survival compared with the vehicle-treated mice (Fig. 8a). PDX58 showed de novo resistance to FGFRi.

Cisplatin is one of the first-line standard-of-care chemotherapeutic agents for EC patients with metastatic disease or at high risk of recurrence, however, molecular subtyping of tumours collected in the PORTEC-3 clinical trial showed that chemotherapy had significant clinical benefit only in patients with p53abn tumours^8^. FGFR inhibition has been shown to sensitise PDX and cell line xenografts to cisplatin in lung, ovarian and cervical cancers^29–31^. We, therefore, aimed to assess if the combination might also be more effective in both p53abn and MMRd tumours. We retreated 2 of the BGJ398 treated models as well as 3 additional PDX models with pemigatinib and cisplatin alone and in combination (Fig. 8b, c). All three PDX models representing the MMRd molecular subtype (PDX52, PDX59 and PDX67) showed some effect on TGI but no effect on overall survival with cisplatin alone, and no additional survival benefit was seen in the combination compared to FGFRi alone (Fig. 8b). In the PDX models representing the p53abn subtype of EC (Fig. 8c), we found a variable response. PDX23 has wildtype p53 by sequencing but represents serous EC with a copy number high genomic profile^24^. Although significant TGI was seen in PDX23 with each treatment, only the combination of pemigatinib with cisplatin significantly prolonged survival. In contrast, PDX61 showed a significant improvement in survival with FGFRi, but there was no additional benefit with the addition of cisplatin (Fig. 8c). Treatment-related toxicity or weight reduction were not observed in mice treated with FGFRi alone however, 95% of the mice treated with both FGFRi and cisplatin often presented with ruffled fur potentially due to cisplatin toxicity.

For the PDX models treated with BGJ398, tumour morphology and tumour growth characteristics were compared between the vehicle-treated (tumour collected when the final volume of 900mm^3^ was reached) and the residual tumour that regrew after completion of treatment (collected at the survival endpoint when the tumour reached ~900mm^3^). Treated PDX tumours demonstrated significant central necrosis, cystic formation and tumour differentiation that were not evident in the tumours from mice in the vehicle-treated control arm consistent with the PDXOs findings (Supplementary Fig. 9).

**Fig. 9:**
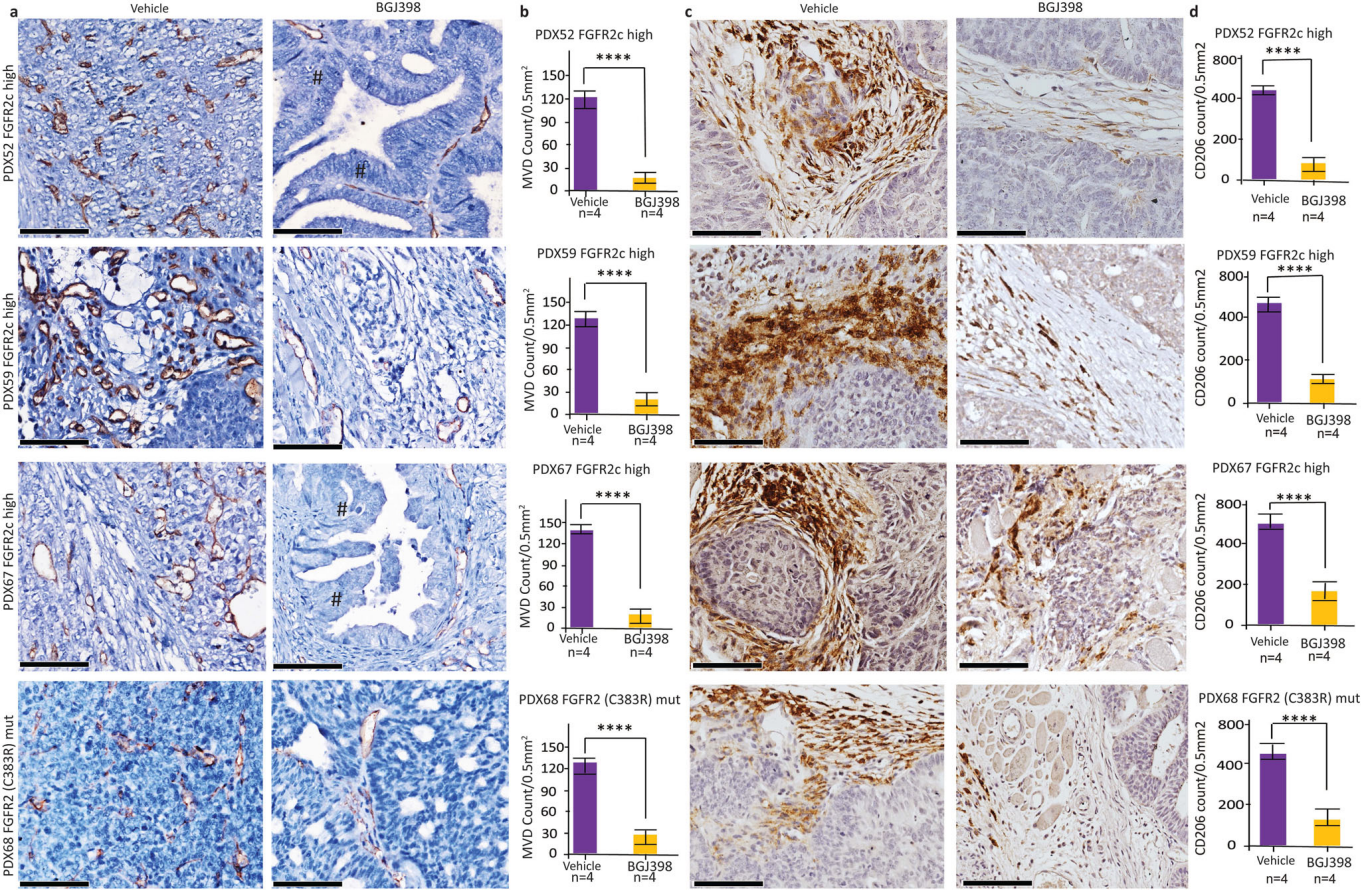
FGFR inhibitor significantly reduced tumour microvessel density (MVD) and CD206 + M2 Macrophages in EC PDXs with FGFR2 dysregulation. **a** Representative microphotography images of CD31 IHC stain on tumours from four independent PDX models treated for 7 days with either vehicle (left panel) or BGJ398 (right panel) **b** Quantification of CD31+ microvessel density (MVD) in BGJ398 versus vehicle-treated tumours for each indicated model. **c** Representative microphotography images of CD206 + M2 polarised Macrophages in tumours from four independent PDX models treated for 7 days with either vehicle (left panel) or BGJ398 (right panel) **d** Quantification of CD206 + M2 polarised macrophages in BGJ398 versus vehicle-treated tumours for each indicated model. PDX68 carries mutationally activated FGFR2 (C383R) while PDX52, PDX59 and PDX67 express *FGFR2c*. MVD and CD206 were counted using automated image analyses (Qu Path) and the score was reported as an average count of MVD or CD206+ cells/0.5mm^2^. Significant differences were assessed with a two-sided student’s T-test (**** *P* < 0.0001) and error bars indicate standard error of the mean (SEM), Scale bar 50 µm. # areas of differentiation with glandular formation.

To assess the impact of FGFRi on tumour proliferation and tumour immune microenvironment, 4 independent PDX models were treated with 30 mg/Kg BGJ398 for 7 days and quantified several biomarkers in control and treated tumours. PDX68 carries mutationally activated FGFR2 and was therefore included as a positive control to determine if similar effects were seen in models with FGFR2c isoform switching. As expected, a significant reduction in expression of Ki67 was observed in BGJ398 treated compared to vehicle-treated tumours for all four models (Supplementary Fig. 10a, b), consistent with our in vitro findings. A statistically significant (P < 0.0001) reduction in microvessel density (MVD) that was quantified using IHC CD31 staining was noted in BGJ398 treated PDXs compared to vehicle-treated tumours (Fig. 9a, b). Studies in other cancer models have reported that the FGF/FGFR signalling pathway modulates several different subsets of immune cells within the tumour microenvironment, including myeloid derived suppresser cells (MDSC) and M2 tumour-associated macrophages via FACs^32,33^. The study also confirmed a significant reduction in CD206 expressing macrophages (M2) following 7 day BGJ398 treatment in both PDX68 (FGFR2 mutant) and the three EC PDX models with FGFR2c activation (Fig. 9c, d). We also noted 7 days of BGJ398 treatment enhanced the differentiation of EC, with clear evidence of glandular morphology formation after treatment compared with poorly differentiated solid morphology in PDX52 and to a lesser extent PDX67 in vehicle-treated mice as indicated areas with **#** in Fig. 9a.

## Discussion

Women with advanced EC have limited treatment options and currently available treatment modalities are suboptimal. Development of effective precision-targeted therapies requires authentic preclinical models that represent the morphological and molecular profiles of the patient tumours with high fidelity. In this study, we report the establishment and characterisation of PDXOs from authentic PDX models which represent the aggressive histologic and molecular subtypes of ECs where improved treatments are urgently needed. We have also presented robust functional data showing FGFR2c oncogenic dependence of ECs and FGFR2c is a new opportunity for targeted therapy that can be evaluated in a clinical trial.

The success rate of PDXOs establishment and expansion in this study was very high (94%) compared to the previously published reports (22%-40%) from primary patient tumours^27,28^. The high success rate in our study likely reflects the selection of more aggressive tumours in our original panel of PDXs, and potentially an increased enrichment for cancer stem cells during PDX passaging. Our established PDXs and PDXOs predominantly represent G3 ECs of different histological types and include the four molecular subtypes of EC that are clinically relevant for drug discovery and testing. The PDXOs are very stable and retain the morphological and molecular characteristics of original parental donor tumours after cryopreservation and/or serial passaging (P15). This ensures the potential utility of our PDXOs for future drug or drug combination screening to optimise precision therapy of EC patients. We also showed that both the carcinomatous and sarcomatous component in the UCS models could be retained when the PDXO was initially cultured in partial osteogenic media^25^ compared to epithelial stem cell media.

Initial screening of the primary patient tumours, PDXs and matched PDXOs for FGFR2 isoform status revealed similar patterns of FGFR2c splice isoform expression to that reported in a large clinical cohort of EC patients^14^. This data supports the use of these PDXOs and PDXs as authentic preclinical models that can be used to assess if FGFR2c expression is a therapeutic target in EC.

FGF2 is an empirical growth factor component of stem cell media and is indicated to be a principal master regulator of cancer stem cells^34^, pluripotent adult stem cells, and embryonic stem cells^35,36^. In contrast to other reported studies where FGF2/FGFR signalling has been reported to be critical for the growth and self-renewal of colon cancer PDOs^37^, we report that the FGFR2c expressing PDXO models were not reliant on the inclusion of FGF2, EGF or the WNT3A, Noggin and R-Spondin supplements in the stem cell media. This contrasts with previously reported growth factor withdrawal experiments in EC PDOs where EGF and R-Spondin were reported to be required for optimal growth^28^. This finding suggests heterogeneity of EC PDXOs growth factors requirements, presumably representing the heterogeneity in oncogenic pathways altered across the various models. Different growth factor requirements for PDOs depending on the activation of different oncogenic pathways have been reported previously for colon cancer PDOs^37,38^.

We have shown that PDXOs with FGFR2c expression were dependent on endogenous FGF2 for growth/proliferation and self-renewal, as the inclusion of the FGF2-specific neutralising antibody or 100 nM BGJ398 24 h after initial organoid seeding resulted in a significant reduction in PDXO number and size. To validate autocrine FGF2-driven activation of FGFR2c in EC, we used a specific in situ PLA to show receptor phosphorylation and correlated this with endogenous FGF2 by IF. In the ASPDX67-CL cells, we observed high FGFR2c phosphorylation but only when cells were cultured in 10% FBS and confirmed a reduction in expression of endogenous FGF2 in serum-starved conditions which correlated with the reduction in receptor phosphorylation. In the PDXO67 organoids, we also showed high FGFR2c phosphorylation, even when FGF2 was specifically excluded from the media. The importance of autocrine FGF2 expression in activating FGFR2c in EC is supported by the identification of high FGF2 expression in all 7 models assessed. This is in contrast to the paracrine activation of FGFR2b that occurs in normal epithelial cells^12^. It should be noted that there is redundancy in FGF/FGFR signalling, and FGFR2c could also be activated in EC by additional ligands including FGF1, FGF9 and FGF18.

Morphological analyses of well-established PDXOs at 72 h showed a higher BGJ398 concentration (300 nM) was required to inhibit the proliferation and induce organoid degeneration when they were mature and well-established. This is a higher concentration than we have previously reported for FGFR2-mutated EC cell lines grown in 2D^20^. We then proceeded to show in vitro treatment of a panel of PDXOs with 300 nM BGJ398 led to cell death in the three PDXOs expressing FGFR2c and not in the two EC PDXOs with low/no expression. We were specifically interested in assessing if FGFR2c isoform switching alone resulted in oncogene dependence and confirm that FGFRi would induce cell death. To demonstrate this, we used LIVE/DEAD^TM^ assay accompanied by high-content automated image analyses that simultaneously assess morphological features, proliferation/cytostatic status, and cytotoxic effects of drugs compared to ATP-based proliferation assays. To confirm that FGFR2c is a valid target of BGJ398 in these models and not FGFR1 or FGFR3, we then performed lentiviral mediated shRNA knockdown of *FGFR2* in four PDXO models. Similar results were shown with two independent shRNAs in *FGFR2c* expressing EC PDXO models, but not in the PDXO53 with very low *FGFR2c* expression, indicating that the effect of BGJ398 was indeed due to the inhibition of FGFR2c.

To confirm that FGFR2c expression was a valid therapeutic target in EC, 5 PDX models were treated in vivo with clinically relevant doses of BGJ398 (30 mg/Kg) or Pemigatinib (1 mg/Kg). Both BGJ398 and Pemigatinib demonstrated significant tumour growth inhibition in 4/5 models expressing FGFR2c. This initial 21-day treatment also led to a significant doubling of survival for these four sensitive models. PDX68 carries a similar mutation to the JHUEM2 cell line (FGFR2^C383R^) with known in vivo sensitivity to BGJ398^20^. Although more TGI was seen in PDX68, significant improvements in TGI as well as overall survival were also seen in the FGFR2c expressing PDX models, confirming FGFR2c expression in the absence of an activating mutation as a therapeutic target. The mechanism of resistance in PDX58 is currently unknown. The PDX58 tumour that was reimplanted for the current drug study was propagated from a sequenced tumour at F0 generation with no obvious activation of a parallel or downstream signalling pathway. Work is ongoing in the laboratory to identify the mechanism of resistance in PDX58.

The morphologic assessment of sensitive tumours that regrew when treatment was stopped showed significant central necrosis and cystic formation with a small residual rim of tumour cells evident. This indicates gross tumour volume measured with callipers immediately following FGFRi treatment did not correspond to tumour burden. This observation has clinical relevance regarding the type of imaging used to assess tumour shrinkage and treatment response in patients in the clinic and suggests that CT morphological criteria or other functional imaging would be superior to RECIST size-based criteria, to more accurately reflect viable tumour burden.

As FGFR inhibition has been shown to sensitise ovarian and cervix cancer cell lines and xenografts to cisplatin^29–31^, we aimed to assess if the combination might be more effective in p53abn PDXs and MMRd PDXs. Our findings showed that although cisplatin had some effect on TGI during the initial 21d treatment, neither cisplatin alone nor the addition of cisplatin to pemigatinib had any benefit on survival in three independent MMRd PDXs with FGFR2c expression consistent with PORTEC 3 clinical trial finding^8^. This data raises the possibility that patients with MMRd ECs their tumour harbouring oncogenic FGFR2 activation by isoform switching may not benefit from the recently reported chemotherapy plus immunotherapy combinations^39,40^ due to intrinsic chemotherapy resistance. However, this hypothesis obviously requires validation in patient samples treated with chemotherapy plus immunotherapy. In the two PDX models representing p53abn EC with moderate or high FGFR2c expression, we also showed significantly improved survival with either FGFRi alone (PDX61) or the combination of cisplatin and FGFRi (PDX23). Our data indicate that in the context of FGFR2 activation in EC, maybe FGFRi is chemosensitising only a subset of patients with p53abn EC. Additional work to identify the mechanism underlying the improved survival seen in PDX23 with the cisplatin + FGFRi combination is required. This data also suggests that while FGFR2c expression was not prognostic in p53abn EC patients, potentially underpowered due to a small sample size^14^, the inclusion of FGFRi in future combination therapies may provide clinical benefit to a subset of patients in the second-line cisplatin-resistant setting.

Assessment of tumour cell proliferation (Ki67) and MVD (CD31) via IHC in tumours from 4 different models treated with BGJ398 for 7 days showed that BGJ398 treatment resulted in not only a significant reduction in cancer cell proliferation but also a significant reduction in MVD. Importantly these results were similar between the three FGFR2c expressing PDX models and PDX68 representing FGFR2 mutant EC. Previous studies in other FGFR-dependent breast and lung cancer models have shown that FGFRi reduces angiogenesis^32^. Analysis of expression changes in three *FGFR2* mutant EC cell lines treated with the FGFRi Debio1347 for 24 h (GSE73024)^41^ shows a reduction in expression of several angiogenic growth factors including VEGFA (>2 fold), CTGF (3–18 fold) and GDF15 (2–6 fold). We therefore, hypothesize that the MVD reduction observed in our FGFR2c EC PDX models is due to both the reduction in expression of pro-angiogenic growth factors downstream of activated FGFR2 as well as direct inhibition of FGFRs expressed by endothelial cells. A higher microvessel density has also been associated with poor prognosis and shorter survival in EC^42,43^. We also showed that BGJ398 treatment resulted in enhanced differentiation in some PDX models, consistent with the role of FGFR2c in promoting epithelial-mesenchymal transition and dedifferentiation of cancer cells. This suggests it may be another mechanism by which FGFR inhibition exerts its anti-tumour activity in ECs with FGFR2c expression.

In other cancer types, FGFR dysregulation plays a significant role in tumour immune evasion and immunosuppression and importantly FGFRis have been shown to synergise to increase the efficacy of immune check inhibitors (ICI)^33,44,45^. A significant reduction in pro-tumour CD206 + M2 polarised macrophages in all four EC PDX models confirmed that FGFR inhibition plays a significant role in modulation of the immune TME in these EC models. In EC a high number of tumour-associated macrophages has also been reported to be associated with disease progression and poor clinicopathologic biomarkers^46,47^. M2-macrophages are reported to contribute fetal-maternal immune tolerance during embryogenesis^48^ and the endometrial tumour cells may share the same pathway for immune evasion. The combination of pembrolizumab and lenvatinib has recently been approved for all EC subtypes, however, a very high proportion of patients (~90%) develop toxicity requiring dose reduction and complete responses are low. In hepatocellular cancer PDX models, treatment with 20 mg/kg BGJ398 for 14 days has been shown to result in significantly improved vascular normalisation as quantitated by IHC markers and lectin perfusion^49^. Based on this, we propose that FGFRi may synergise with ICI due to the combination of tumour cell killing, improved vascular normalisation and blocking of tumour-intrinsic immunosuppression mechanisms. A Phase II clinical trial of FGFRi + PD-1i is ongoing in EC patients with *FGFR2* mutations/fusions (NCT04463771). Our data suggest more EC patients may benefit with the inclusion of patients with high/moderate FGFR2c expression in future trials of similar combinations.

In conclusion, we have developed and characterised EC PDXOs that recapitulate the morphological and molecular profile of advanced ECs and their corresponding PDX models. We have identified authentic preclinical models with different patterns of *FGFR2c* oncogenic splice isoform expression and showed that PDXOs with high FGFR2c expression are dependent on endogenous FGF2 for its autocrine activation. Finally, the study revealed PDXOs and matched PDXs with both moderate and high FGFR2c expression are highly sensitive to FGFRi in vitro and in vivo. Collectively the data presented confirm the expression of the FGFR2c splice isoform as a precision predictive biomarker in EC and supports the initiation of phase II clinical trial testing the combination of an FGFRi with ICI in the metastatic setting.

## Methods

### Patients, tumour Implantation, PDX expansion and characterization

The study was conducted according to the Declaration of Helsinki. Tumours were collected from EC patients following Human Research Ethics Committee (HREC) approval (HREC/15/MHS/127) and QUT HREC (#1500000169, 1500000323). All participants provided written informed consent that their excess tissue could be used for future HREC-approved research projects. Ethical clearance for laboratory animal use was granted from the UQ Animal Ethics Committee (AEC) (UQ/TRI/021/19) and QUT (1900000701). We have previously established 18 EC PDXs with different histological and molecular subtypes^24^. Under QUT HREC approval (#5194), we have also re-established three PDXs provided by the Antonio Gil-Moreno Laboratory at the Vall Hebron Institute of Research (VHIR) in Spain initially developed under ethics approval (PR(AMI) 276/2018). Early passage (F2/F3) PDXs were implanted and during passaging portions of the tumour were frozen for later DNA/RNA extraction, preparation of formalin-fixed paraffin-embedded (FFPE) tumour blocks to assess for morphology and FGFR2 expression via IHC/RNA ISH, as well as placed in freezing medium (10% DMSO in FBS) for subsequent organoid culture or PDX expansion (Supplementary Fig. 1).

Fresh surgical resected tumours either from primary or distant metastatic sites were collected from patients who had surgery under patient informed consent. Tumour was collected using RPMI (Thermo Fisher Scientific, VIC, Australia) transport medium with 100 IU/ml penicillin and 100 mg/ml streptomycin antibiotic (Thermo Fisher Scientific, VIC, Australia) and placed on ice in double contained cooler and transported to the lab for implantation. The tumour was dissected using a surgical blade and segregated as depicted in Supplementary Fig. 1. Approximately 1–2 mm fragment of tumours embedded with 50 µL GFR extracellular matrix 50% Matrigel^TM^ (Cat# 354230, Corning, NY, USA) were implanted subcutaneously into immunocompromised Nod Scid Gamma (NSG) 8-week female mice as described previously^24^. In brief, Tumour implantation was performed under anaesthesia with Isoflurane vaporizer with 4% at induction and 2% during maintenance and maintaining oxygen flow at 1 L/min. In addition, analgesic (buprenorphine (temgesic) 0.3 mg/ml) 0.075 mg/Kg) using an insulin microsyringe with 29 G attached needle was administrated subcutaneously 20 min prior to anaesthetic induction. Following anaesthesia, a small (0.5 mm) subcutaneous incision under the shoulder blade was performed and the tumour fragment was inserted in the pocket of the incision and closed with tissue adhesive (Vetbond). The mouse was placed in a prewarmed cage and monitored through the recovery period. When the PDX tumour reached the AEC-approved target volume of 900mm^3^, mice were euthanized using medical-grade compressed CO_2_ with a fill rate of 40-50% of the chamber volume/min. Serial passaging of the PDXs was carried out as described above. Each tumour was collected under a sterile technique when reached the target volume following euthanasia with CO2. Approximately ~1-2 mm fresh fragment tumour mixed with 50 µL Matrigel^TM^ was transplanted into each recipient mice as required for subsequent passaging and a portion of the tumour was frozen with 90% FBS and 10% DMSO for later transplantation (biobanking) as outlined in Supplementary Fig. 1. Portions of the tumour were also frozen for DNA/RNA and protein extraction, fixed in formalin and embedded in paraffin (FFPE) to assess for morphology and FGFR2 expression via IHC/RNA ISH as well as placed in media for subsequent organoid culture (Supplementary Fig. 1) when applicable. Genomic characterization (WES, WGS) was performed in 11 PDXs as published previously^24^.

IHC to assess FGFR2, p53, ER, PR, Ki67 expression, MMR protein status and RNA ISH was performed in matched patient tumours, PDXs and PDXOs at different passages (P2-10) to confirm PDXOs recapitulated PDX models.

### EC PDX derived organoids (PDXOs) establishment and expansion

Fresh PDX tumours were collected following a sterile procedure, dissected and a portion of the tumour was placed in RPMI medium containing antibiotics (penicillin/streptomycin) and antimycotic and transported on ice to the tissue culture (TC) room. PDX tumours were minced utilising a surgical blade and dissociated into clusters of tumour cells by mechanical dissociation and enzymatic degradation of the extracellular matrix using the Human Tumor Dissociation Kit (Cat#130-095-929, Miltenyl Biotech, VIC, Australia) following the manufacturer’s protocol. The dissociated tumour clusters in suspension were centrifuged, and the pellet was resuspended in DMEM/F12 medium containing 2 mmol/L L-glutamine with antibiotics (100 µg/ml Promicin (cat# amp-1, Jomar life Research, VIC, Australia), 100 U/mL penicillin and 100 mg/mL streptomycin). The suspension of tumour cells was then filtered through a 70 µm cell strainer (Biostrategy, VIC, Australia) to remove large tumour stroma clusters. The tumour cluster cells passed out through the strainer were pelleted and resuspended in cold organoid media with constituents reported previously^50^ and were plated into the centre of a 48 well plate precoated with 50% V/V) growth factor reduced (GFR) Matrigel^TM^ (cat# 354230, Corning, VIC, Australia) and incubated at 37 °C and 5% CO_2_. When Matrigel^TM^ was gelled, 200 µL of warm organoid media^50^ was added to cover Matrigel droplets and the media was changed every 3 days.

Our initial organoid media was enriched with 5% conditioned media for Noggin and R-Spondin in addition to the other supplements^50^. As our PDXOs were established from aggressive grade 3 ECs and to tailor their GFs requirements, the recipe of our organoid media was simplified. The modified organoid media thereafter is referred to in this paper as standard organoid media. This medium contained advanced DMEM/F12 (basal medium) with 1% HEPES buffer, 2 mmol/L L-glutamine, antibiotics (100 µg/ml Promicin (cat# amp-1, Jomar life Research, VIC, Australia), 100 U/mL penicillin and 100 mg/mL streptomycin) and 10 µM Y-27632 ROCK inhibitor (Selleck Chemicals Inc) enriched with the following recombinant human GFs 50 ng/ml EGF, 10 ng/ml FGF2, 10 ng/ml WNT3A, 100 ng/ml Noggin, 250 ng/ml R-Spondin-3. We noted a similar growth and morphology of established PDXOs as well as the successful establishment and propagation of multiple new PDXOs in our standard organoid media. We have also used this latter media to successfully expand the PDXOs that were established in our initial organoid medium^50^. All GF withdrawal experiments in PDXO59, PDXO67 and PDXO56 and functional studies were performed in standard organoid media. Based on the withdrawal experimental results, we have cultured and passaged PDXOs in this standard organoid media after the removal of some or all growth factors and the addition of 10% FBS, indicating aggressive EC PDXO cultures do not require specialised organoid media.

PDXOs growth was monitored using an inverted phase contrast/light microscope and images were captured to monitor growth using a Nikon Ts2U inverted microscope attached to the camera and computer with NIS-Elements software. Established PDXOs were further propagated and passaged and were subjected to multiple sequential cycles of freezing and thawing to ensure the renewal stability and fitness of the organoids. PDXOs were banked at multiple passages by cryopreservation in 10% DMSO/90% FBS in liquid nitrogen. For the establishment of the primary PDX-derived EC cell line, dissociated cells were cultured in 2D in a 10 cm petri dish in DMEM/F12 with 10% FBS, penicillin and streptomycin. Cancer cell selection was performed with differential trypsinisation (0.25% trypsin EDTA) to remove fibroblasts. After 4 successive passages, 100% purity was confirmed with a cytokeratin 7 stain. Mycoplasma testing was performed routinely using a commercial kit (MycoSEQ and Thermo-Fisher Scientific, VIC, Australia).

### PDXOs formalin-fixed paraffin-embedded (FFPE) blocks preparation

The medium was removed and replaced with a cold medium, and the plate was cooled on ice to liquefy the Matrigel to make PDXOs suspension. The PDXOs in suspension were gently transferred into a yellow screw cap, flat base 5 ml Polypropylene tube (Sarstedt, Ref.# 60.9921.524) and kept at room temperature (RT) for 20 min, centrifuged at 300 g at 4 °C for 4 min, the supernatant discarded and the PDXO pellets were washed with PBS twice and fixed with 4% PFA in PBS for 1 h at RT. Then PDXO pellets were centrifuged at 300 g for 4 min at RT and the fixative was discarded. A 2% Histogel/agarose gel (Cat. # HG-4000-012, Thermo Scientific, VIC, Australia) in PBS was prepared and melted by heating in the microwave and then cooled to 60 °C prior to adding to the PDXO pellets. Melted agarose gel (200 µL) was added to PDXO pellets, allowed to solidify at RT for 30 min or 10 min at -20 °C, then carefully transferred to tissue cassettes with a sponge to avoid loss of small PDXOs, placed in 70% ethanol and processed using an automated 4 h processing protocol at the TRI-Histology core facility and embedded with paraffin to make FFPE blocks. H/E and IHC staining and BaseScope RNA ISH assay were performed as described previously^14,51^.

### FGFR2 isoform determination using BaseScope RNA ISH

FGFR2b and FGFR2c splice isoform expression on primary EC patients and matched PDXs and PDXOs tumours was determined following our previously optimised and validated BaseScope RNA ISH assay^14^. In Brief, 4 µm tissue thickness sections were cut, deparaffinised, dehydrated, and target retrieval was performed using a pressure cooker (Decloaker Chamber, Biomedcare, CA, USA) with 10 mM of Na acetate at PH 6. A human-specific, exon 7-8 junction target probe (BA-Hs-FGFR2-tv2-E7E8, NM_022970.3, 1578-1622 bp, 1ZZ, Cat# 715151) and an exon 7-9 junction specific probe (BA-Hs-FGFR2-tv1-E7E9, NM_000141.4, 1580-1619bp, 1ZZ, Cat# 710031) were deployed to target to the FGFR2b and FGFR2c isoforms respectively. Peptidylprolyl isomerase B (PPIB) a housekeeping gene probe (BA-Hs PPIB, Cat# 710171) was used as a positive control. All probes and BaseScope reagents were purchased from (Advanced Cell Diagnostic (ACD) Bio, Hayward, CA, USA) unless otherwise stated. Each probe was incubated for 2 h at 40°C oven and serial amplification was performed according to the optimised protocol and detection was performed using FAST RED chromogen (ACD BIO, Hayward, CA, USA). Finally, the section was counterstained with Mayer haematoxylin and mounted using aqueous mounting medium and scoring was performed as described in the Fig. 2 legend.

### Molecular subtyping

Molecular subtyping of the EC patient, PDX and matched PDOs tumours were performed using the ProMisE algorithm combining genomic analyses (tumour mutation burden, somatic copy number alteration) or IHC surrogate markers as previously reported^3^. IHC was done for several markers, including p53, MLH1, MSH2, MSH6 and PMS2, ER and PR, FGF2, FGFR2, FGFR1 and FGFR3 either using diagnostic automated Ventana or manual protocols described below.

### Ligand stimulation of primary cell line and PDXOs

The ASPDX67-CL primary cell line at passage 4 was cultured in DMEM/F12 media with 10% FBS and after reaching 70% confluence, they were trypsinised and 1 ×10^5^ cells were seeded in 6 well plates containing 3 coverslip slides/well (1.13 mm Ø) with 10% FBS media for 48 h at 37 °C and 5% v/v CO_2_. After cells reached 60–70% confluence, media was removed, and cells were washed with warm DPBS and either incubated with fresh 10% FBS media or starved overnight (16 hr) with 0.5% FBS DMEM/F12 media. At zero time point (T0) media was removed and washed with warm DPBS twice and stimulated with media containing 0.5% FBS with 5 µg/ml HS with or without 10 ng/ml FGF2 and incubated for 30 min at 37 °C and 5% CO_2_. For the treatment arm, 300 nM BGJ398 was added during stimulation. Cells were then washed with DPBS twice and fixed with 4% Paraformaldehyde (PFA) in PBS for 30 min at room temperature (RT) and PLA was performed. For PDXOs, culturing was performed in standard organoid medium without growth factors and 30% (V/V) Matrigel^TM^ as described above and at Day 10 media and Matrigel was removed and washed with DPBS and fresh standard organoid medium without growth factors +/- 5 µg/ml HS + 10 ng/ml FGF2 and incubated for 30 min. For the treatment group, 300 nM BGJ398 was added during ligand stimulation. After 30 min, the media was removed and PDXOs were fixed with PFA for 1 h and resuspended and stained with Immunofluorescence (IF) or Proximity Ligation Assay (PLA) assay as described below.

### Immunofluorescence (IF) and proximity ligation assay (PLA) staining

Immunofluorescence and Proximity Ligation Assay (PLA) staining was performed manually using optimised and validated protocols. In brief, culture media was removed and cells and/or organoids were washed with PBS and fixed with 4% paraformaldehyde (PFA) with incubation for 30 min. PFA was removed and cells/organoids were washed three times with PBS/TPBS and permeabilised with 0.25% TritonX100 in PBS for 5 min at RT and washed with PBS/ TBS three times. Cells were blocked with 2% BSA in PBS for 1 hr at RT or overnight at 4 °C. After that, cells were transferred into a humidity chamber and incubated with primary antibody diluted with 2% BSA in PBS (Supplementary Table S2 for respective antibody source and dilution) and incubated for 90 min at room temperature or overnight at 4 °C. Slides were then washed three times with TBS or PBS for 5 min each and incubated with secondary antibodies (Supplementary Table S3 for dilutions and sources) for 45 min at RT. For PLA staining, primary antibodies (antiFGFR2 and pFGFR653) were incubated for 90 min after 3 times wash with TBS, cells/PDXOs were incubated for 90 min with PLA probes (oligonucleotide conjugated donkey anti-rabbit, PLUS (DUO82002) and donkey anti-mouse, MINUS (DUO82004)). After 3x washes with TBS, the ligation and amplification steps were followed according to the manufacturer protocol (detail guideline suppl method A). To combine PLA with IF, the primary antibody was incubated for 60 min at 37 °C after the amplification step and then washed 3 times with TPS for 2 min each. Then, the secondary antibody conjugated with FITC was incubated for 30 min at 37°C and washed with TPS twice for 2 min each. Finally, cells/organoids were washed two times with PBS or TBS for five min each and the final wash was performed with MilliQ H_2_O to remove any precipitated salt and mounted with ProLong Gold Antifade with DAPI mounting medium (Thermo-Fisher, VIC, Australia). PLA signals were quantified in each treatment by taking 5 independent images at 20 airy objective microscope from each of 3 coverslips and these images were analysed using automated Fuji Image J2 where red PLA signals were quantified alongside total cell number using DAPI nuclear co-stain for ASPDX67-CL or Histone-3 co-stain for PDXO67.

### In vitro treatment of PDXOs with BGJ398 (FGFR1-3) inhibitor

To test the effect of FGFR inhibitors in PDXOs regeneration and growth inhibition, PDXOs were initially treated at an early time point following seeding (after 24 hr) with 100 nM BGJ398 (Selleck Chemical, Houston, USA) or at a later time point when the organoids were well established (after 10 days) with different concentrations of BGJ398 (100 nM, 200 nM and 300 nM) at different time points (24 hr, 48 hr and 72 hr) cultured with standard organoid media containing GFs and 50% V/V GFR Matrigel^TM^ After optimising the dose and duration of treatment, three independent PDXOs with high FGFR2c expression and two with negative or low FGFR2c expression) were grown for 10 days. After PDXOs reached 70-80% confluency, PDXOs were treated with either 300 nM infigratinib/BGJ398 (Selleck Chemicals Inc, VIC, Australia) or vehicle (equal volume of DMSO) and incubated for 72 h at 37 °C and 5% CO_2._ The viability of the PDXOs was then assessed using a LIVE/DEAD^TM^ assay kit (Cat# R3760, Thermo Fisher Scientific, VIC, Australia) following the manufacturer’s protocol. This experiment was performed in 3 different PDXO models derived from each PDX as well as in technical triplicate. Images were captured XYZ stack using an inverted rotatory confocal fluorescent laser microscope (FV1200) at 10x objective magnification view airy. Image analyses to quantify the live and dead cells were performed using Fiji ImageJ2 (National Institute of Health, Bethesda, Maryland, USA) as described previously^52^. In brief, the captured images were converted into TIF format and imported for Fiji ImageJ and the image of PDXOs were imported to the Fiji ImageJ2 platform. For each channel (green, red, and blue) and Otsu’s threshold was used to create binary images (white and black) and the level of pixel intensity was assigned between 1 (white) and 0 (black), the object was identified using region of interest (ROI) and analysis set in parallel sequential. The total number of cells for each channel (live=green) and (red=dead) were calculated as follows; the number of green positive cells divided by the number of nuclei (DAPI positive), likewise, the number of red positive cells divided by the total number of nuclei. The results were exported to SPSS for statistical analysis.

### shRNA mediated FGFR2 knockdown in endometrial cancer PDXOs

*FGFR2* knockdown was performed using short hairpin RNA (shRNA) targeting two different exons (exon 2 and exon 16) as described in our previous publication with slight modification for organoid application^53^. In brief, two independent pLKO.1 *FGFR2* shRNA constructs targeting exon 2 (TRC clone ID TRCN0000000367) and exon 16 (TRC clone ID TRCN0000218493) were purchased from Sigma Adrich (St Louis, MO, USA) which have been shown to successfully knockdown *FGFR2* in previous studies^54^. The nucleotide target sequences of these shRNAs are provided in Supplementary Table 4. A third *FGFR2* non-silencing/non-target (NT) shRNA (5′-TTCTCCGAACGTGTCACGT-3) was used as a negative control. The plasmid preparation and lentiviral production were conducted as described previously^53^. In brief, each target plasmid was transduced into HEK293FT cells (sourced from ATCC, Manassas, VA, USA) along with packaging vectors (pNHP, pTAT, pHEV-VAVG). First, retroviral packaging cell line (HEC293 FT cells) was prepared by seeding∼5 × 10^6^ cells in T75 flask with 15 ml of DMEM/10% FBS containing selective antibiotics. The following day, medium was removed and replaced with fresh media without antibiotics. The transfection mixture was prepared by adding lipofectamine 2000, Opti-Mem, packing plasmid and the lentiviral shRNA constructs and was combined with the DNA mixture in 15 ml tube and incubated for 20 min at RT. This transfection mixture was added drop-by-drop to HEC293 FT cells when reached 80-90% confluency and incubated at 37 °C. After 24 h, media was removed and replaced with fresh media and incubated for another 24 h and lentiviral stocks were harvested at 48 h post-transfection. PDXOs were cultured in standard organoid media without GFs Matrigel+ 10% FBS. Lentiviral transduction of EC organoids was performed according to published protocols with some amendments^55^. In brief, four independent PDXO52, PDXO59 and PDXO67 with FGFR2c high expression and PDXO53 without *FGFR2c* expression (biological control) were cultured in standard organoid media for 10 days and after well recovered from their frozen state, viability was checked using Live/Dead assay. After 10 days from the date of seeding the organoids became 80% confluent and the medium was taken and replaced with 400 µL fresh ice-cold standard organoid media to each well and the media with organoids were collected into precooled 15 ml Falcon tubes (FT). The organoids were centrifuged at 300 g at 4 °C for 5 min, media suspension was discarded, and organoids were resuspended in 500uL of fresh media and broken into small clusters by pipetting with 100 µL tip several times, centrifuged and pelleted. The pelleted organoids were resuspended with precooled lentiviral suspension of the respective shRNAs (exon 2, 16 and NT) in a 1:1 ratio with standard organoid media in 50% Matrigel (25% Matrigel final), thoroughly mixed and seeded into 24 wells ultra-low attachment (ULA) (Corning CLS3473) plate and incubated at 37 °C, 5% CO_2_ for 24 h. Following removal of media containing lentivirus, 400uL cold media was added to each well, the contents were transferred into a 15 ml FT, centrifuged at 300x g for 3 min and the organoids were pelleted and washed in cold PBS 3 times. Finally, the organoid pellet was resuspended in standard organoid media without GFs + 10% FBS with 50% Matrigel and seeded into 24 well ULA plate (Corning CLS3473) and incubated at 37 °C, 5% CO_2_ for 1 hr. After the Matrigel had solidified, 300uL prewarmed organoid media was added and organoids were monitored for growth using a light microscope attached to a camera (Nikon). Media was changed every 3 days and 0.5 µg/mL Puromycin was added on day 4 for selection. On day 10 viability was checked using the Live/Dead assay and high-content image analyses were performed as described above under the section of in vitro treatment of PDXOs. At this timepoint, a portion of the organoids were pelleted, fixed in 4%PFA for 2 h and organoid blocks were prepared as detailed in the above section and FGFR2c BaseScope RNA ISH was performed to evaluate the extent of FGFR2 knockdown as described above. FGFR2c expression was used as a surrogate of FGFR2 for 3 reasons; 1) the organoids predominantly express FGFR2c, 2) FGFR2c is the proto-oncogenic isoform 3) BaseScope is more specific and indicates spatial expression and can be performed more effectively on smaller samples. PPIB a housekeeping gene was used as a positive control to show the relative expression of FGFR2c in the PDXOs. The experiment was performed in technical triplicate and independent biological triplicate.

### Western blot for FGF2 antibody validation

BaF3 cells (primarily sourced from ATCC, Manassas, VA, USA) were previously transduced with FGF1, FGF2, FGF3, 7-10, 17, 18, 20 and 22 ligands tagged with Myc-DDK^TM^ (OriGene, USA). Protein extraction and western blot analysis were performed as previously published^20^. Twenty micrograms of protein lysates were loaded from BaF3 cell lysates expressing targeted ligands and probed with anti-FGF1, anti-FGF2, anti-FGF3 and anti-FGF7 antibodies (see Supplementary Tables 2 and 3 for source and cat#) to assess potential antibody -cross reactivity. Cell lysates were probed with an anti-Myc antibody to demonstrate that each cell line was expressing the transduced FGF ligands. The most relevant raw uncropped WB gels are available in Supplementary Fig. 5c.

### In Vivo treatment of PDXs

The experiment was conducted according to the approved protocols AEC (#021/19, 399/20, 456/20) and the Australian National Health Medical Research Council (NHMRC) guidelines. EC tumour fragments were implanted into 6-8-week female NSG mice subcutaneously under a sterile technique for each model. A 1-2 mm PDX tumour fragment collected from F2/F3 regrown model was engrafted subcutaneously into an 8-week female NSG mouse for each PDX model. When the tumour reached 150-200mm^3^ mice were randomized into vehicle, 30 mg/kg infigratinib/BGJ398 (Selleck Chemicals) (3 models), 1 mg/Kg pemigatinib (Incyte) alone (7 models), pemigatinib + cisplatin (5 models), or cisplatin alone (5 models). Two PDX models (PDX52 and PDX59) were treated with both infigratinib and pemigatinib. Mice were treated for 21 days with infigratinib and pemigatinib via oral gavage and 5 mg/Kg cisplatin intravenous (IV) injection weekly. Tumours were measured using a digital calliper 3 times/week until reaching 900mm^3^. Tumour volume (TV) was calculated as follows: Tumour Volume (*TV)=Length[width* x *width]*^2^*/2*. Follow-up data regarding tumour size was collected 3x/w until tumours reached 900mm^3^. For tumour biomarkers assessment and mechanistic studies, a short period (7 days) of treatment was aimed to compare apparently equal tumour volume. For this aim, a separate cohort of 4 independent PDX tumour models were treated with BGJ398 or vehicle (4 mice per arm) for 7 days. Mice were euthanised after 6 hr of the last treatment dose and their tumours were collected, cryopreserved or fixed with 4% paraformaldehyde for H/E morphologic examination and IHC biomarker evaluation.

### Immunohistochemistry and quantification

IHC staining of p53 and MMR proteins was performed via Ventana automated stainer (Ventana Medical Systems, Tucson, AZ, USA) using the diagnostic antibodies at Mater Pathology, Brisbane Australia. Detailed information regarding these antibodies’ sources and cat# are provided in Supplementary Tables 2 and 3. The remaining IHC staining was performed manually following optimised protocols. In brief, whole FFPE tumour tissue sections were cut (4 µM thickness) and sections were dewaxed, hydrated and washed, and endogenous H_2_O_2_ was quenched with 3% H_2_O_2_ for 10 min. Antigen retrieval was performed in 10 mM citrate buffer (PH = 6.0) with Decloaking Chamber™ (BIOCARE MEDICAL, Brisbane, Australia). Respective Biotin (Jackson Immunology Labs) or polymer-conjugated secondary antibodies for all primary antibodies raised in rabbit or mouse (K406311-2, DAKO, Envision kit, Agilent Technologies, VIC, Australia) were used. Detailed information for all primary and secondary antibodies sources, Cat#, and period of incubation are provided in Supplementary Tables S2 and S3. Chromogen development was performed using DAB (K3468, DAKO Envision) by incubating for 2-5 min and slides then counter stained with Mayer Haematoxylin (Sigma Aldrich).

P53 and MMR proteins (MLH1, MSH2, MSH6 and PMS2) IHC scoring was performed as previously reported^3^. Quantification of other IHC/IF markers was performed using either automated Fiji ImageJ2 or QuPath bioimage analyses software following the guidelines as published previously^52,56^. In brief, whole section stained slides were scanned using a whole slides scanner (3DHISTECH) at 40X magnification and images in the form of TIF files were imported to either ImageJ2 or QuPath software as indicated to create separate projects for each biomarker of interest or H/E stain. Quantification was performed after training and setting the required annotation, deconvolution and DAB vector intensity for each biomarker based on the algorithms and workflow of Fiji Image J2 or QuPath platform (https://github.com/qupath/qupath)^57^ Results were exported into SPSS or GraphPad Prism as required for data analyses.

### Statistical analysis

Statistical data analyses were performed using SPSS version 23 and GraphPad Prism ver 9.2 as required. Figures annotation. formatting and visualization were performed using Adobe Illustrator (AI). Pearson’s (Chi X^2^) test was used to evaluate correlations between categorical variables. Student T-Test (two-sided) was used to assess continuous variables. Two-way ANOVA was used to assess tumour growth inhibition. For some models, one or more PDX tumour measurements were missing for a time point, in which case a mixed model within PRISM was used to assess tumour growth inhibition. Survival outcome differences between or among groups were computed using the Kaplan Meier Curve with the Mantel-Cox (Log-Rank) test of probability. Multiple comparisons were corrected using either Bonferroni for categorical variables or the Tukey test for continuous variables. A Geisser Greenhouse correction was used for unequal variance. One-way ANOVA with Dunnett’s multiple comparison test was used to compare treatments vs control. *P* value < 0.05 (two sided) was considered statistically significant.

### Supplementary information

Supplementary Information

## Data Availability

The data generated in this study are included in the main article and its supplementary data. The raw genome dataset of WES and SNP arrays supporting the study are deposited in the European-Genome Phenome Archive under study number EGAS00001004666. PDX and PDXO tumour models and other relevant data are available upon reasonable request from the corresponding authors and subject to university policy and ethics approval.

## Code availability

The code required to reproduce the genomic data in this manuscript can be found at https://github.com/okon/EC_PDX_genomics. The code used to perform alignment to human and mouse genome references is available on https://github.com/ampatchlab/nf-pdx.
