# Supplementary material for: Endometrial cancer PDX-derived organoids (PDXOs) and PDXs with FGFR2c isoform expression are sensitive to FGFR inhibition
Source: NPJ Precis Oncol. 2023 Dec 8;7:127. doi: 10.1038/s41698-023-00478-6 (PMC10703877; doi:10.1038/s41698-023-00478-6)
Supplement: Supplementary file 1 — Supplementary Information [file 41698_2023_478_MOESM1_ESM.pdf]

Supplementary Data

Supplementary Table 1: Clinicopathological and Survival outcome data of a cohort of endometrial cancer patients who donated tumours to successfully establish PDXs.

PDX ID	PDXO ID	Age	Histologic type	FIGO Grade	LVSI status	Myometrial invasion	FIGO Stage	Recurrence status Y/N	Survival status D/L (Days)
PDX3	Failed	60	UCS	3	yes	>50%	IC	Y	D (DSS 824)
PDX12	PDXO12	70	EEC	3	No	<50%	IA	N	L (OS 1941)
PDX20	PDXO20	75	Serous EC	3	yes	>50%	IIIC1	Y	D (DSS 601)
PDX21	PDXO21	67	EEC	3	No	>50%	IIIC1	Y	D (DSS 604)
PDX23	PDXO23	58	Serous EC	3	Yes	>50%	IIIB	Y	D (DSS 1883)
PDX24	PDXO24	62	EEC	3	No	>50%	II	LFU	LFU
PDX45	PDXO45	60	EEC	3	yes	>50	II	LFU	LFU
PDX48	NA	61	DEC	3	No	<50%	IA	N	D (DSS 472)
PDX49	PDXO49	70	UCS	3	No	<50%	IA	N	L (OS 1878)
PDX52	PDXO52	83	EEC	2	No	>50%	IB	Y	D (DSS 584)
PDX53	PDXO53	43	[a]EEC	3	No	>50%	II	N	L (OS 1930)
PDX56	PDXO56	79	UCS	3	Yes	>50%	IA	Y	L (OS 1990)
PDX58	PDXO58	62	[b]EEC	3	Yes	>50%	IB	Y	L (OS 1889
PDX59	PDXO59	71	EEC	3	Yes	>50	IB	N	D (OS 77)
PDX60	PDXO60	61	EEC	3	Yes	>50	IIIA	Y	D (DSS 929)
PDX61	PDXO61	65	DEC	3	Yes	>50	IIIC	Y	D (DSS 272)
PDX67	PDXO67	62	EEC	2	No	>50%	II	N	D (DSS 876)
PDX68[#]	NA	56	EEC	3		>50	Lung Met	Y	D (DSS 378)
PDX69	NA	68	EEC	3	No	>50%	IB	LFU	LFU
PDX70	NA	79	UCS	3	No	>50%	IIIA	Y	LFU
PDX71	PDXO71	58	CCC	3	Yes	>50%	IIIC	Y	D (OS 437)

[a] This patient's tumour had a 10% clear cell component; [b] this patient's tumour had a 10% serous morphological component; # PDX was established from a recurrent distant metastatic site (Lung), NA, Not attempted; D/L, Dead/Live vital status, DEC, Dedifferentiated endometrial cancer; EEC, Endometrioid Endometrial Cancer; FIGO, International Federation Gynaecological Oncology; LFU, Lost to follow-up; LVSI, Lymphovascular space invasion; Met, Metastasis, N, No; PID, Patient Identification; PDX ID, Patient-Derived Xenograft Identification; OS Overall survival, DSS, Disease-specific survival, RFS, Recurrence Free survival; Y, Yes

Supplementary Table 2: List of primary antibodies used in the project, supplier/source, Cat# and working protocol.

Primary Antibody	Clone/immunogen	Host	Specificity	Cat# (supplier)	Stock conc. mg/ml	Working dilution	Antigen retrieval and application
Anti-FGFR2	C-17	Rabbit	Human Rat	122 (Santa Cruz)	0.2	1:100, 90 min at RT or 60 min 37ºC 1:200, 4ºC o/n	PC 110ºC, 15Ps for 15 min
Anti-FGFR2	AA621-724	Mouse	Human Rat	Ab58201 (Abcam)	0.1	1:100, 90 min at RT or 60 min 37ºC, 1:200 4ºC o/n	PC 110ºC, 15Ps for 15 min
Anti-phospho pan FGFR653/4	55H2	Mouse or Rabbit	Human Rat mouse	3476 (Cell signalling)	0.1	1:100, 90 min at RT 60 min 37ºC WB 1:1000, at 4 ºC o/n	NA (IF, PLA)
Anti-pERK1/2	P44/42	Rabbit	Human Mouse	137FS	0.1	1:500, at 4 ºC o/n	NA (IF)
Anti-pSTAT3		Rabbit	Human, mouse	9131 Cell Signalling	0.1	1:500 at 4 ºC o/n	IF
Anti-FGF1	Phe16-Asp155	Goat	Human Mouse	AF232 (R&D)	0.1	1:100 90 min at RT 60 min 37ºC WB 1:1000, at 4 ºC o/n	PC 121ºC, 15Ps for 10 min
Anti-FGF2	bFM-2	Mouse	Human Mouse	05-118 Upstate Millipore	1	1:300, 90 min at RT 60min 37ºC, 1:500 o/n 4ºC	PC 120ºC, 15Ps for 10 min
Anti-FGF3	254625	Mouse	Human Mouse	MAB1206 (R&D)	0.5	1:300 90 min at RT WB 1:1000, at 4ºC o/n	PC 120ºC 15 Ps for 10 min
Anti-FGF7		Goat	Human, Mouse	AF251 (R&D)	0.25	1:100 90 min at RT 60 min 37ºC	PC 120ºC, 15Ps for 15 min
Anti CD31	RM1006	Rabbit	Human/mouse	Ab281583 (Abcam)	0.2	1:600 at 4ºC o/n	PC 110 for 15 min
Anti-CD206	1400 AA C-terminus to HMR	Rat	Human, Mouse	Ab64693 (Abcam)	1	1:400 at 4ºC o/n or 1:400 at RT 90 min	Pressure cooker 121ºC, 15Ps for 12 min
Anti- MYC	9B11	Mouse	All species	2276 Cell signalling	1	1:1000 at 4 ºC o/n	N/A WB
Anti-Ki67	D2H10	Rabbit	Human Mouse	9027 Cell signalling	0.2	1:500 at 4 ºC o/n 1:1000 for IF	Pressure cooker @110ºC 15 Ps for15 min
Anti-p53	DO-7	Rabbit	Human	(800-2912) Roche Ventana	prediluted	0.5 µg/ml at 4 ºC o/n	Ventana HIAR
Anti-MLH1	M1	Mouse	Human	(760-5091) Roche Ventana	prediluted	1 µg/ml at 4 ºC o/n	Ventana HIAR
Anti-MSH2	(G219-1129	Mouse	Human	(760-509) Roche Ventana	prediluted	20 µg/ml at 4 ºC o/n	Ventana HIAR
Anti-MSH6	SP93	Mouse	Human	(760-5092) Roche Ventana	prediluted	1 µg/ml at 4 ºC o/n	Ventana HIAR
Anti-PMS2	A16-4	Mouse	Human	(760-5094) Roche Ventana	prediluted	1 µg/ml at 4 ºC o/n	Ventana HIAR
Anti-ER	1D5	rabbit	Human	M7047 (DAKO)	1	5 µg/ml at 4 ºC o/n	Pressure cooker @110ºC 15 Ps for15 min
Anti-PR	PgR636	rabbit	Human	M3569 (DAKO)	1	10 µg/ml at 4 ºC o/n	Pressure cooker @110ºC 15 Ps for15 min
Anti-FGFR1	EPR806Y	rabbit	Human, mouse	Ab676464 Abcam	1	5 µg/ml at 4 ºC o/n	Pressure cooker @110ºC 15 Ps for10 min
Anti-FGFR3	C51F2	rabbit	Human	4574 Cell signalling	1	5 µg/ml at 4 ºC o/n	Pressure cooker @110ºC 15 Ps for15 min

NA, not applicable; PC, pressure cooker; o/n, overnight; WB, western blot; MW, Microwave, Ps, Pascal; HIAR, Heat-induced antigen retrieval

Supplementary Table 3 List of secondary antibodies used in the project, supplier/source, Cat# and working protocols.

Secondary antibody	Host	Cat#	supplier	Conc. µg/ml	Working dilution	Purpose
Rhodamine (TRITC) anti-rabbit	Donkey	711-025-153	Jackson Immuno-research	500	1:500	IF
Rhodamine anti-mouse (TRITC)	Donkey	715-025-152	Jackson Immuno-research	500	1:500	IF
Rhodamine (TRITC) anti-rat	Donkey	712-025-150	Jackson Immuno-research	500	1:500	IF
FITC anti-rabbit	Donkey	715-545-151	Jackson Immuno-research	500	1:500	IF
FITC anti-mouse	Donkey	715-545-152	Jackson Immuno-research	500	1:500	IF
HRP anti-mouse	Goat	A445	Sigma	1000	1:5000	WB
HRP- anti-goat	Donkey	A345	Sigma	1000	1:5000	WB
HRP- anti-rabbit	Goat	A0545_1ML	Sigma	1000	1:5000	WB
Biotin-SP anti-rabbit	Donkey	711-065-152	Jackson Immuno-research	500	1:300	IHC
Biotin-SP anti-mouse	Donkey	715-065-151	Jackson Immuno-research	500	1:300	IHC
Biotin-SP anti-rabbit	Donkey	705-065-003	Jackson Immuno-research	500	1:300	IHC
Anti-mouse/rabbit		K406311-2	Agilent DAKO	prediluted	Pre-diluted	IHC

IF, Immunoflourescence; IHC, Immunohistochemistry; WB, western blot,

Supplementary Table 4 The nucleotide target sequence of FGFR2 shRNA mediated knockdown of endometrial cancer patient-derived xenograft organoids (PDXO).

Clone ID	Name	Exon	Primer Sequence
Scramble shRNA	pLKO.1 NT	-	5’-TTCTCCGAACGTGTCACGT-3
TRCN0000218493	FGFR2-493	16	5’-AGCCCTGTTTGATAGAGTATA-3
TRCN0000000367	FGFR2-367	2	5’-GCCACCAACCAAATACCAAAT-3

NT, None targeting

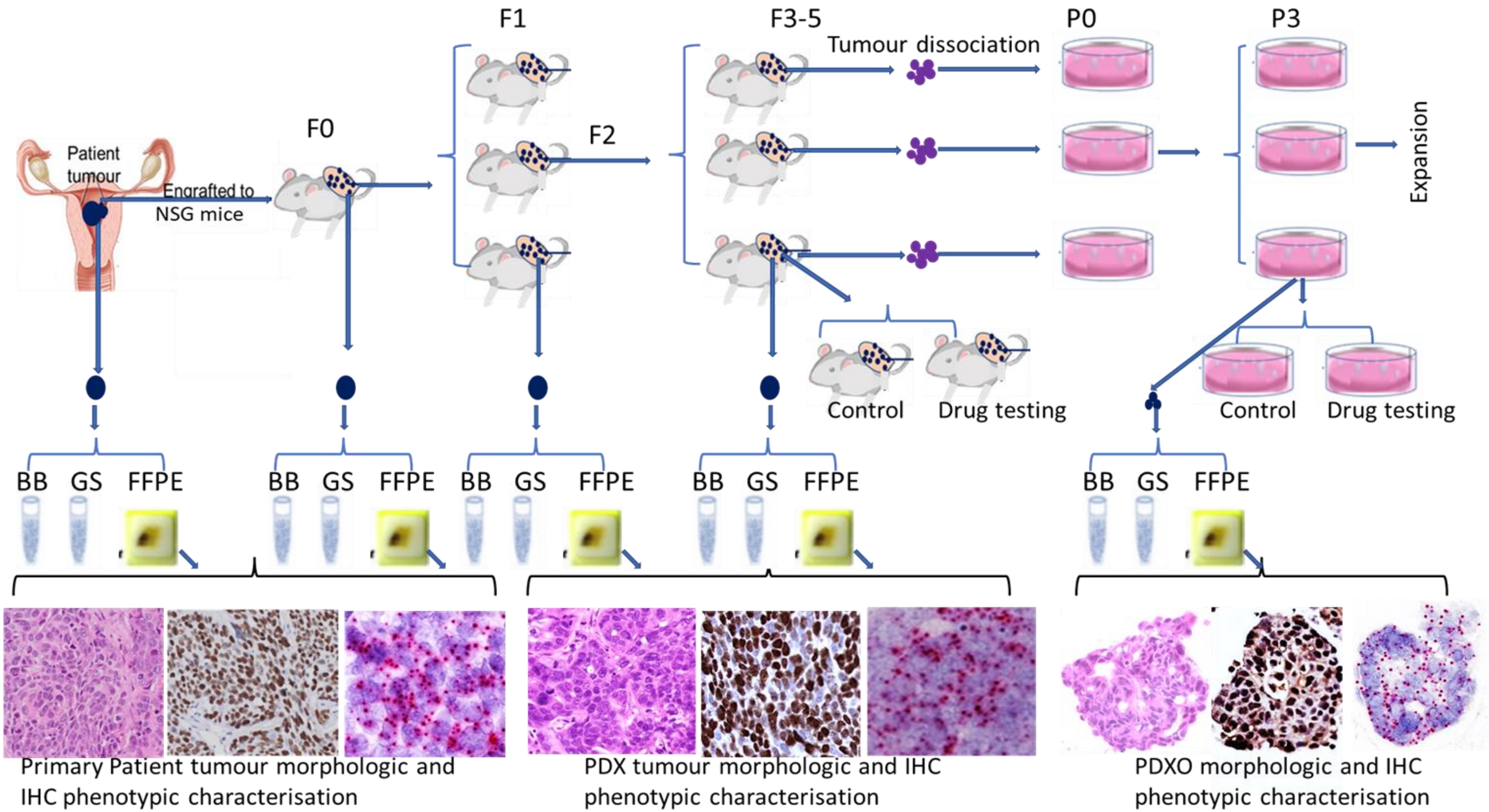
F0
F1
F2
F3-5
P0
P3
Patient tumour
Engrafted to NSG mice
Tumour dissociation
Expansion
Control
Drug testing
Control
Drug testing
BB GS FFPE
BB GS FFPE
BB GS FFPE
BB GS FFPE
BB GS FFPE
Primary Patient tumour morphologic and IHC phenotypic characterisation
PDX tumour morphologic and IHC phenotypic characterisation
PDXO morphologic and IHC phenotypic characterisation

Supplementary Figure 1: Schematic diagram illustrating the research design and workflow of the primary tumour and corresponding PDXs and PDXOs characterisation and drug testing. A small portion of fresh tumour was taken from a hysterectomy sample placed in transport media and implanted into an NSG mouse to establish PDX (F0). Another fresh portion of tumour was taken for biobanking and genomic profiling. A large portion of the tumour sample was formalin-fixed and paraffin-embedded (FFPE) for morphologic and biomarker assessment. Each established PDX was further expanded to have multiple generations for drug testing and organoid development. With a similar approach to the primary patient tumour, each PDX tumour was collected and partitioned for implanting into the next-generation PDX or organoid development (when applicable), biobanking, genome sequencing and FFPE for morphologic and target biomarker characterisation. For ex-vivo experiments, PDX-derived organoids (PDXOs) were developed by taking fresh PDX tumour fragments (from F3-F5 PDX passage) and then culturing them in 3D in vitro. After 3-4 passages, these PDXO samples underwent similar morphologic and biomarker assessment. The representative images are the original patient tumour, PDX and PDXO for PDX67. Drug screening was first performed in PDXOs, and later validated in PDXs. BB, Biobanking; GS, genome or exome sequencing; F0, 1, 2 3, generation 0, 1, 2, 3; FFPE, Formalin Fixed Paraffin Embedded; P0, Passage 0.; IHC, Immunohistochemistry

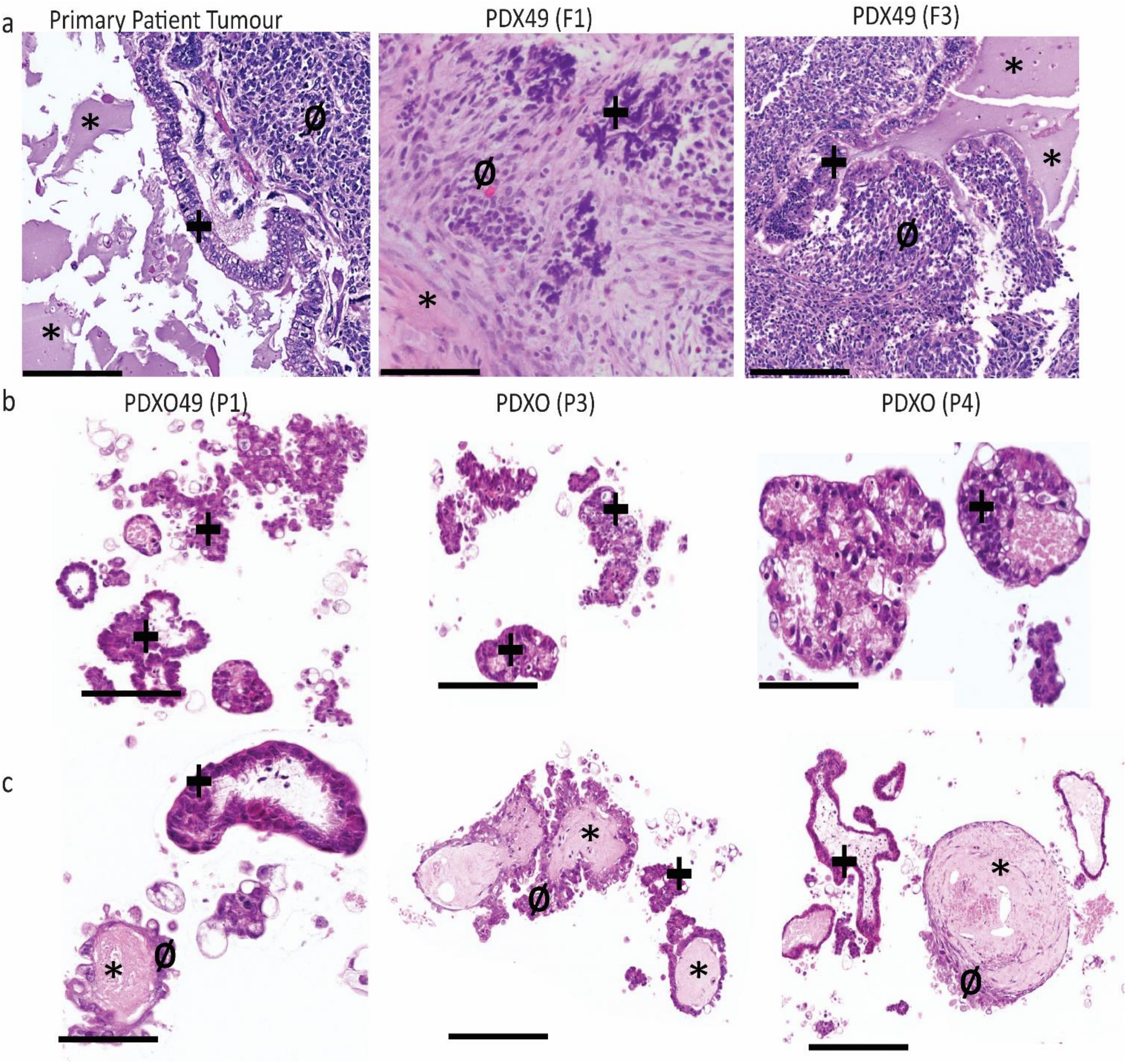

Supplementary Figure 2: Representative Carcinosarcoma PDX and PDXO that recapitulates the primary patient tumour. a) Representative H&E images showing histology of the primary patient tumour and PDX from the indicated passages illustrating the carcinomatous and sarcomatous components that were maintained in the PDX. b-c) Representative images of PDXO49 successfully established from a F3 PDX which maintained only the carcinoma component when grown in standard advanced media including the ROCK inhibitor (b) and both carcinoma and sarcoma components with osteoid differentiation when cultured without ROCK inhibitor and the addition of 10 µg/ml heparin sulphate and 50 µg/ml ascorbic acid (c). Scale bar indicates 100µm. +, carcinoma component; *, osteoid; Ø, sarcomatous component

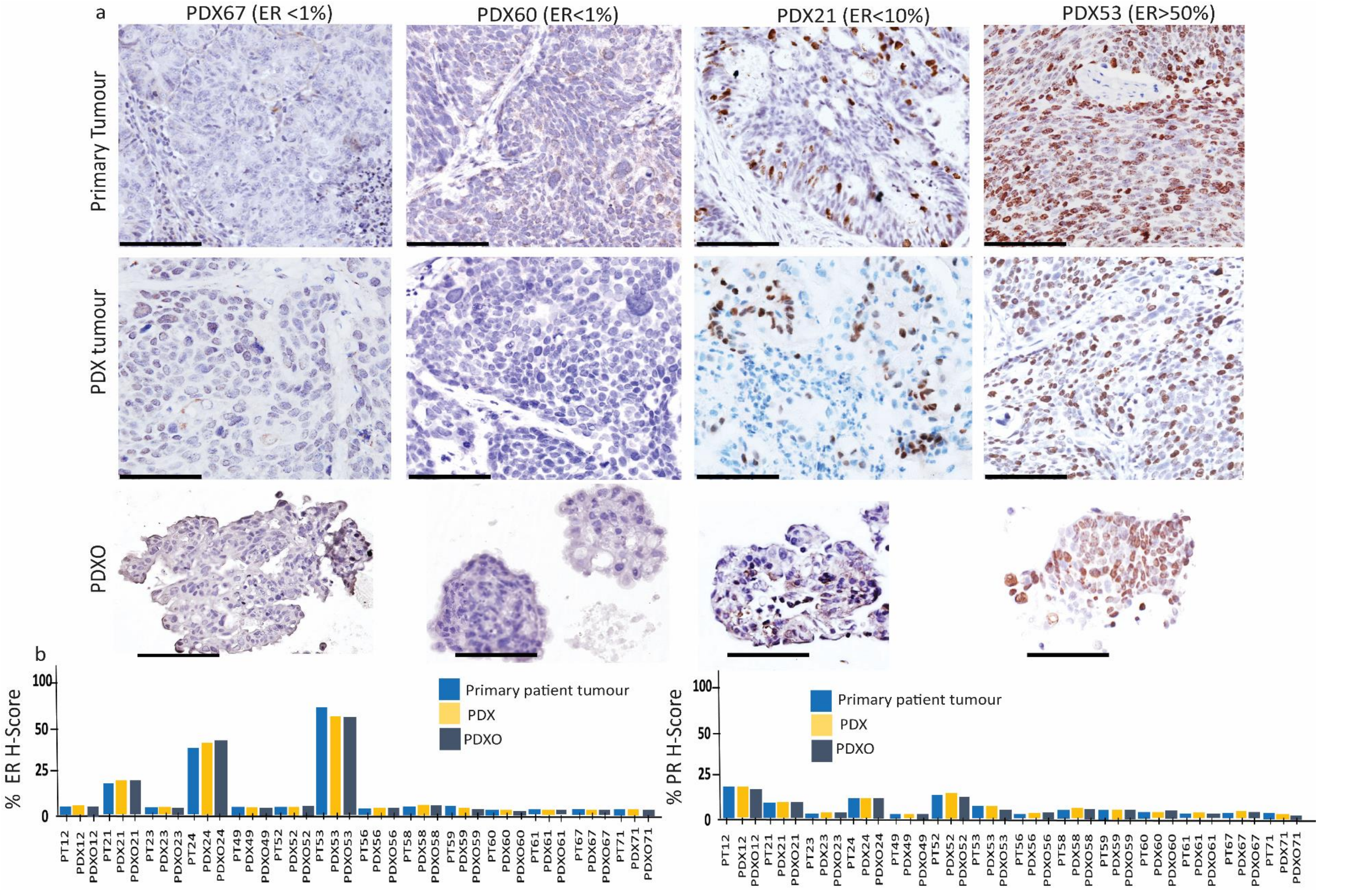

a
PDX67 (ER <1%)
PDX60 (ER<1%)
PDX21 (ER<10%)
PDX53 (ER>50%)
Primary Tumour
PDX tumour
PDXO
b
% ER H-Score
100
50
25
0
Primary patient tumour
PDX
PDXO
PT12 PDX12 PDXO12 PT21 PDX21 PDXO21 PT23 PDX23 PDXO23 PT24 PDX24 PDXO24 PT49 PDX49 PDXO49 PT52 PDX52 PDXO52 PT53 PDX53 PDXO53 PT56 PDX56 PDXO56 PT58 PDX58 PDXO58 PT59 PDX59 PDXO59 PT60 PDX60 PDXO60 PT61 PDX61 PDXO61 PT67 PDX67 PDXO67 PT71 PDX71 PDXO71
% PR H-Score
100
50
25
0
Primary patient tumour
PDX
PDXO
PT12 PDX12 PDXO12 PT21 PDX21 PDXO21 PT23 PDX23 PDXO23 PT24 PDX24 PDXO24 PT49 PDX49 PDXO49 PT52 PDX52 PDXO52 PT53 PDX53 PDXO53 PT56 PDX56 PDXO56 PT58 PDX58 PDXO58 PT59 PDX59 PDXO59 PT60 PDX60 PDXO60 PT61 PDX61 PDXO61 PT67 PDX67 PDXO67 PT71 PDX71 PDXO71

Supplementary Figure 3: Pattern of oestrogen receptor and progesterone receptor expression of endometrial cancer in matched primary patient tumours, PDX and PDXOs. a) representative images of oestrogen expression of primary patient tumours and corresponding indicated PDX tumours and PDXOs. b) H-score of ER and PR in 12 of the primary patient tumours and corresponding PDXs and PDXOs. Scale bar indicates 100µm. ER, oestrogen receptor; PR, Progesterone receptor; PDXs, Patient-derived xenografts; PDXOs, Patient-derived xenografts organoids; H-Score, Histologic Score; PT, Patient primary tumour.

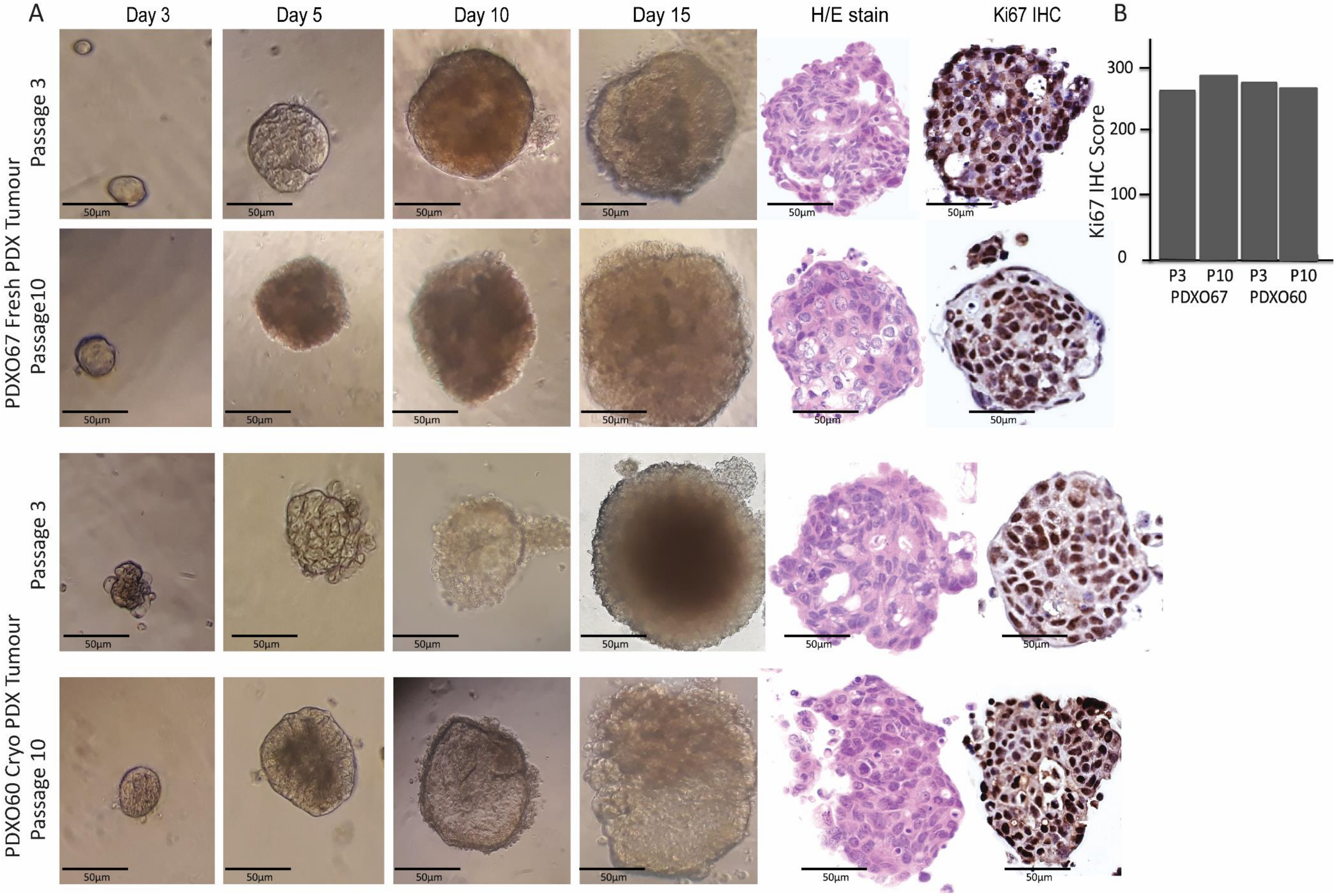

A
Day 3
Day 5
Day 10
Day 15
H/E stain
Ki67 IHC
PDXO67 Fresh PDX Tumour
Passage 3
Passage10
PDXO60 Cryo PDX Tumour
Passage 3
Passage 10
50µm
B
Ki67 IHC Score
300
200
100
0
P3
P10
P3
P10
PDXO67
PDXO60

Supplementary Figure 4: PDXOs maintained their histologic morphology and proliferation capacity throughout sequential passaging.

a) Representative microscopic images, corresponding H/E and Ki67 of PDXO67 established from fresh PDX tumour (top panels) and PDXO60 established from PDX60 tumour fragments that were cryopreserved for 3 years (bottom panels). The PDXOs were cultured at passages (P3) and (P10) for 15 days and tracked at different time points (as indicated). b) Ki67 IHC score in PDXO67 and PDXO60 at passages 3 and 10. Cryo, cryopreserved; H/E Haematoxylin/Eosin; IHC, IHC; Immunohistochemistry. FBS, Foetal bovine serum; GF, Growth factor; Scale bar 50um

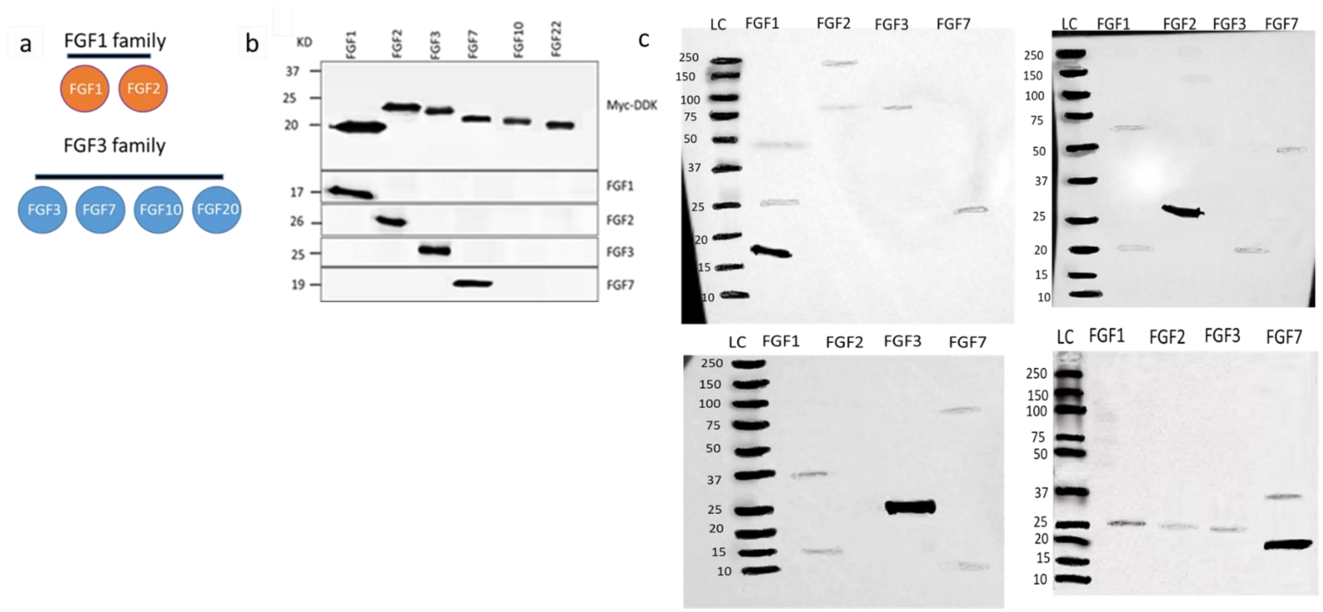

Supplementary Figure 5: Optimization and validation of FGF ligands antibodies.

a) Schematic diagram showing the subfamily clustering of FGF ligands according to their protein homology similarities. b) Western blot analyses of BaF3 cell lines stably transduced with individual FGF ligands to confirm the specificity of antibodies within the FGF1 and FGF3 families. c). Uncropped images of WB gel showing FGF1, FGF2, FGF3 and FGF7. Twenty micrograms of protein lysates were loaded from BaF3 cells expressing ligands and probed with anti-FGF1, FGF2, FGF3 and FGF7 antibodies to assess potential immune-cross reactivity. Cell lysates were probed with an anti-Myc antibody to demonstrate that each cell line was expressing the transduced FGF ligand. Cell lysates were probed with an anti-Myc antibody to demonstrate that each cell line was expressing the transduced FGF ligand. Note, each FGF ligand was performed with separate gel loading and an example of Myc-DDK data is shown.

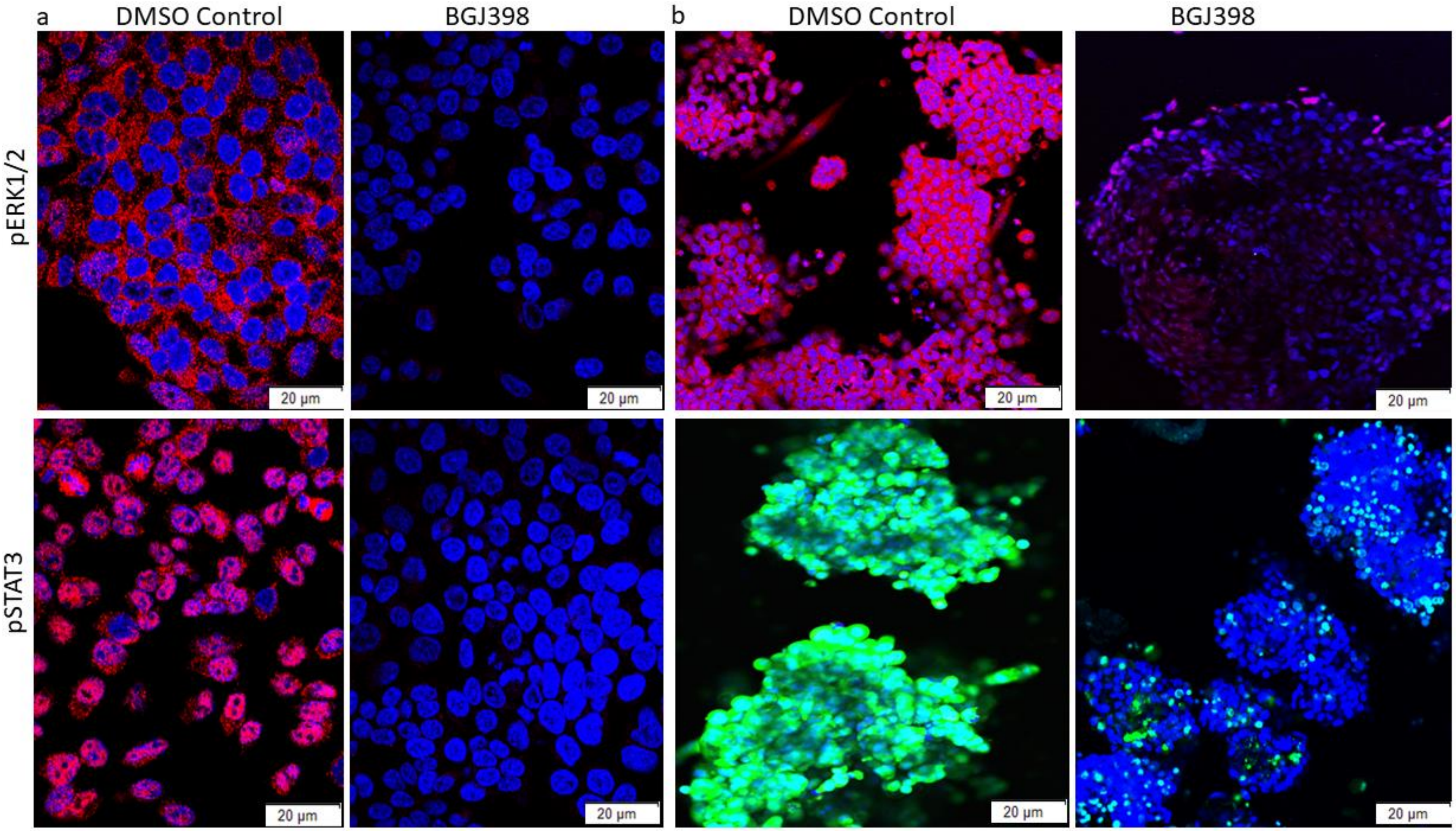

Supplementary Figure 6: Reduction in expression of pERK1/2 and pSTAT3 in BGJ398 treated EC primary cell line and PDXO. Representative images illustrating expression of pERK1/2 (upper panel) and pSTAT3 (lower panel) in DMSO (control) and 300nM/ml BGJ398 treated (a) primary cell line established from PDXO67 (ASPDX67-CL) and b) PDXO67.

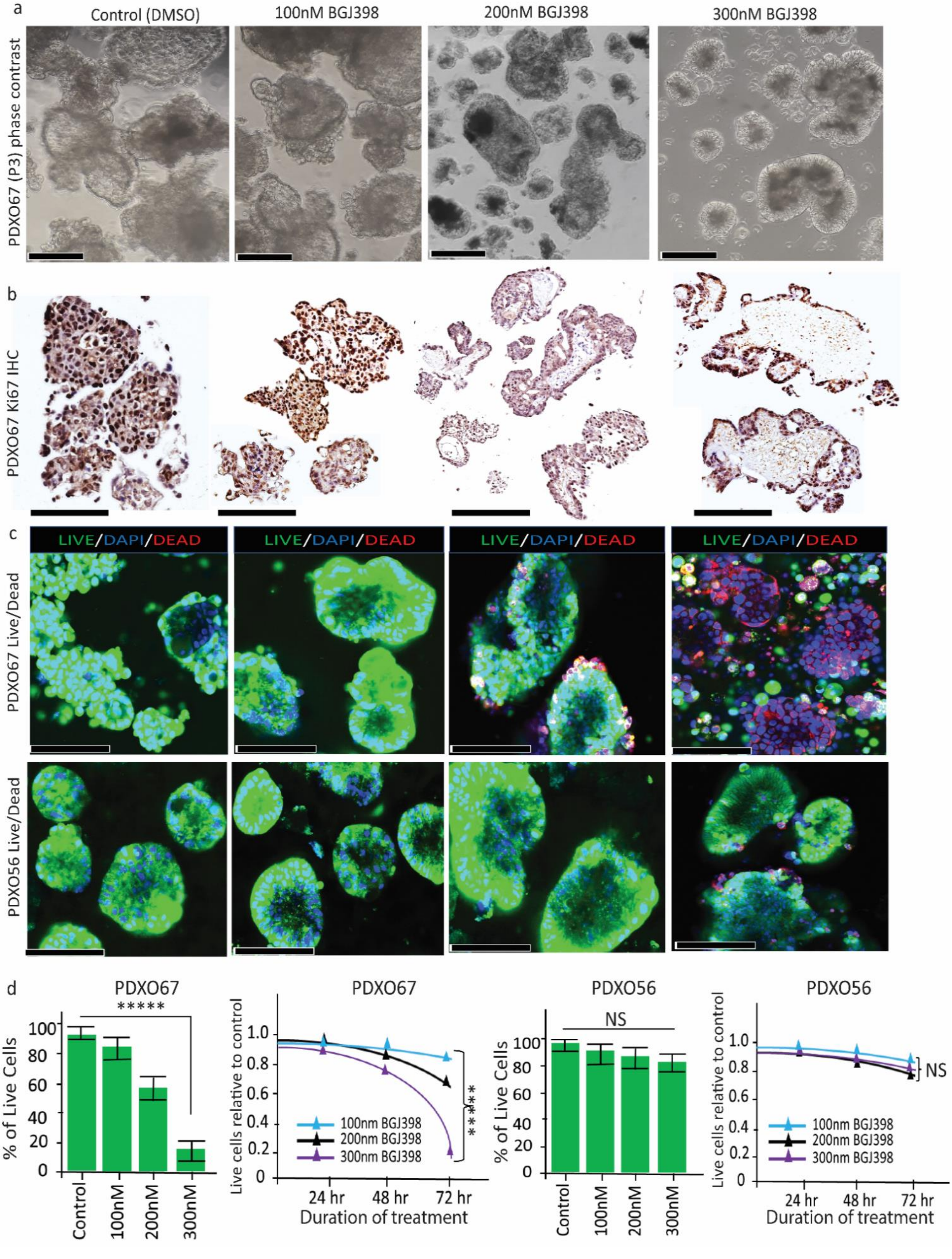
a
Control (DMSO)
100nM BGJ398
200nM BGJ398
300nM BGJ398
PDXO67 (P3) phase contrast
b
PDXO67 Ki67 IHC
c
LIVE/DAPI/DEAD
PDXO67 Live/Dead
PDXO56 Live/Dead
d
PDXO67
*****
% of Live Cells
Control
100nM
200nM
300nM
Live cells relative to control
100nm BGJ398
200nm BGJ398
300nm BGJ398
24 hr
48 hr
72 hr
Duration of treatment
PDXO56
NS

Supplementary Figure 7: The effect of FGFR inhibitor on mature well-established EC PDXOs with three different BGJ398 concentrations and at different time points.

Representative images of mature EC PDXO67 (grown for 10 days before treatment initiation) and treated with different BGJ398 concentrations as indicated for 72 hrs. a) phase contrast microscopy of three-dimensional endometrial cancer organoids b) Ki67 expression on FFPE control and BGJ398 treated organoids c) Representative images of the Live/Dead assay in PDXO67 (FGFR2c high, MMRd) and PDXO56 (FGFR2 negative, p53 abn) treated either with DMSO or three different BGJ388 concentrations as indicated. Green fluorescence shows esterase activity of live cells (Calcein AM), red fluorescence is generated upon binding of Ethidium homodimer-1 to DNA in dead cells and nuclei were stained with DAPI. Images were captured using an inverted laser confocal fluorescent (FV1200) Olympus microscope. d) quantitative analyses showing the proportion of live cells following 72hr treatment with BGJ398 at different doses (bar graph) or at a different time point (line graph) for PDXO67 and PDXO56. The experiment was performed in technical triplicate as well as independent biological triplicates using independent PDXOs established from three different mice carrying each PDX. One-way ANOVA with Dunnett's multiple comparison test was used to compare treatments vs control **** $P<0.0001$. Error bar indicates SEM. The scale bar indicates 200µm. The cell viability of PDXOs was assessed using a LIVE/DEAD assay kit following the supplier’s protocol and quantification was performed using automated Fiji ImageJ2. Ab, Antibody; FGF2, Fibroblast Growth Factor 2; IHC, Immunohistochemistry; PDXO, Patient-Derived Xenograft Organoids. NS not significant

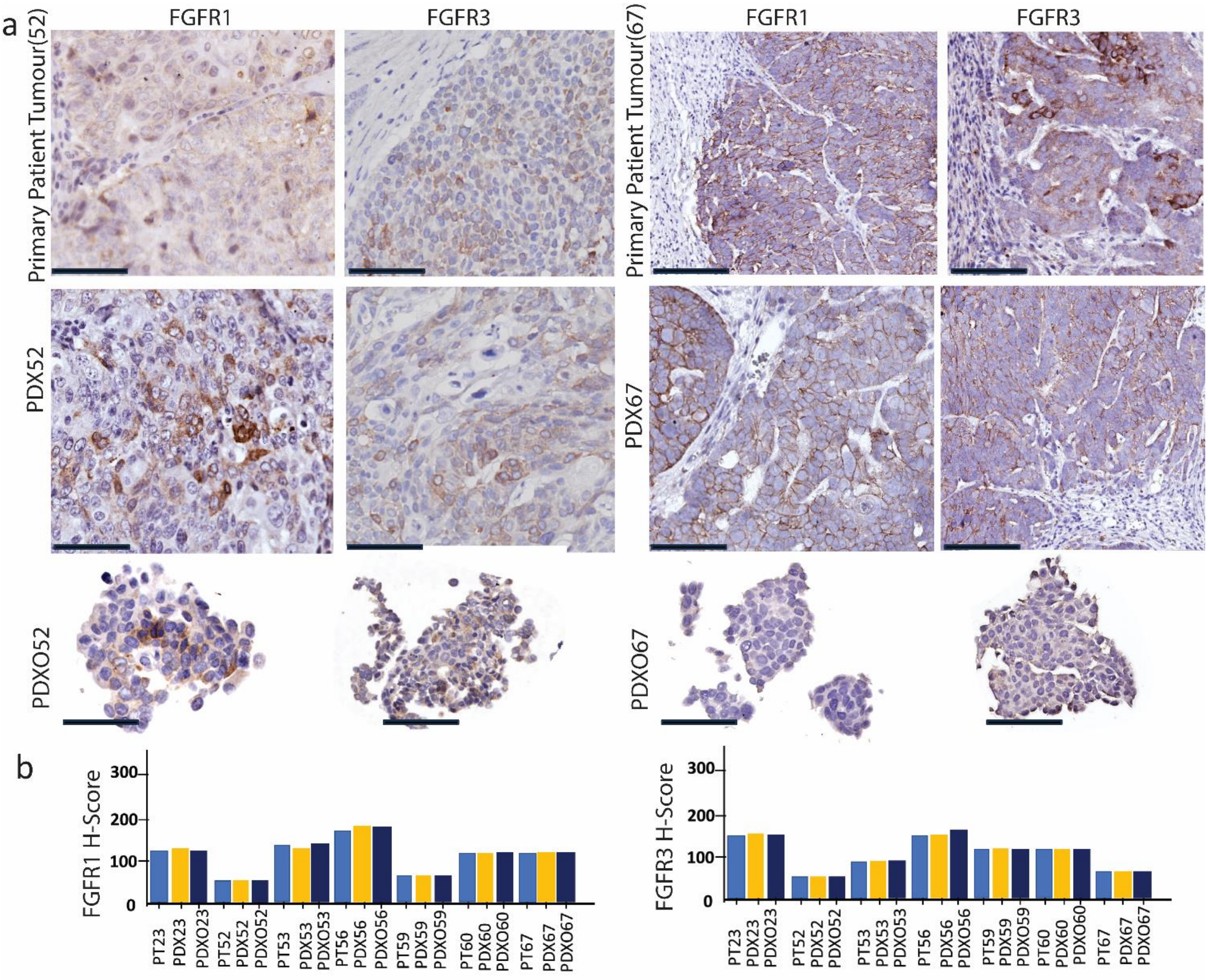

Supplementary Figure 8: FGFR1 and FGFR2 expression in primary patient tumours and corresponding PDXs and PDXOs endometrial cancer. a) Representative immunohistochemistry images showing weak expression of FGFR1 and FGFR3 in indicated patient tumour (top panels) and matched PDXs (middle panels) and PDXOs (bottom panels). b) Histologic score of FGFR1 (left) and FGFR3 (right) of 7 patient tumours and matched PDXs and PDXOs where sufficient tissue sections were obtained for immunohistochemistry assessment. Scale bar indicates 100µm.PDX, patient-derived xenograft; PDXO, Patient-derived xenograft organoids; PT, Patient tumour.

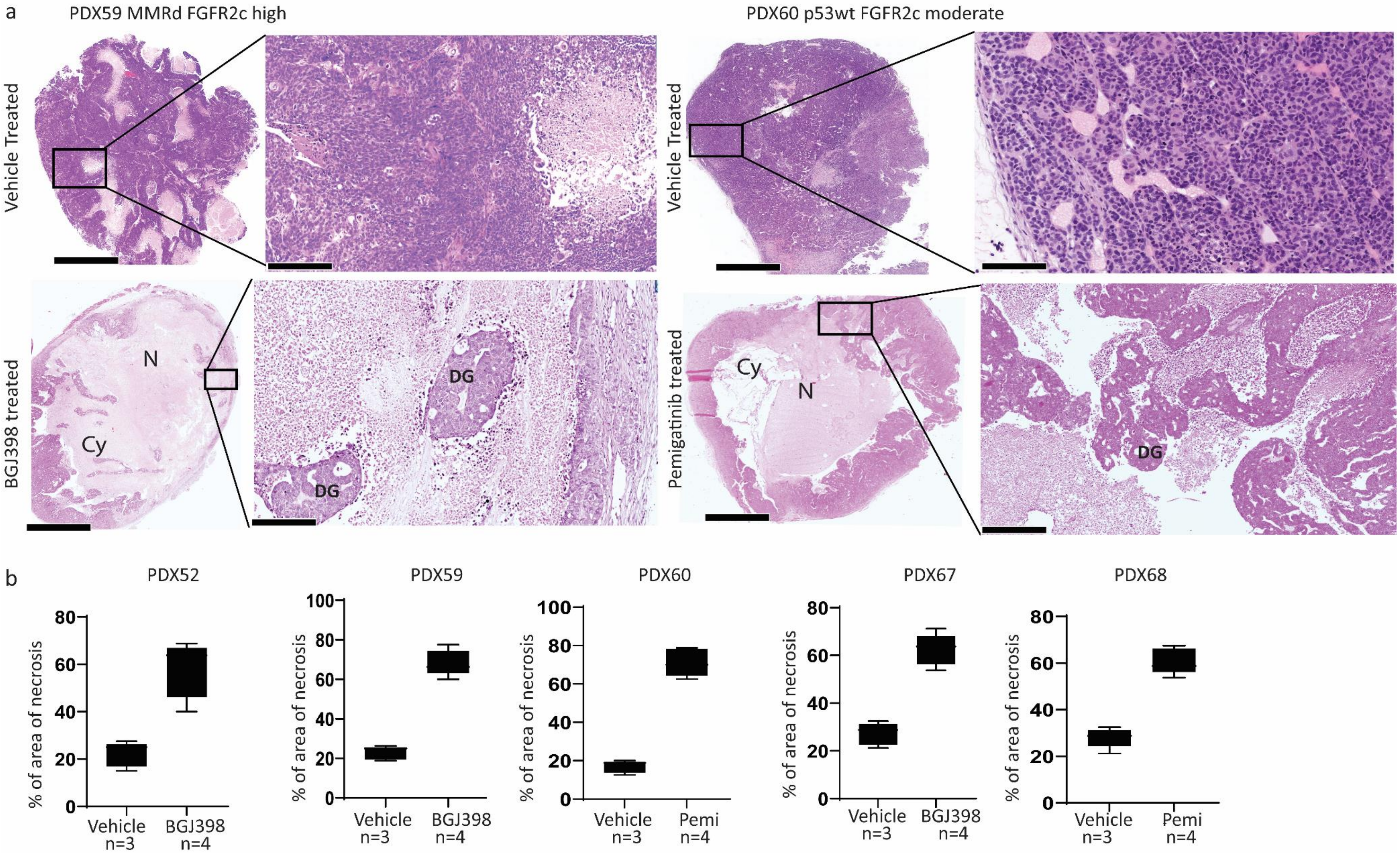
a
PDX59 MMRd FGFR2c high
PDX60 p53wt FGFR2c moderate
Vehicle Treated
BGJ398 treated
Pemigatinib treated
N
Cy
DG
b
PDX52
PDX59
PDX60
PDX67
PDX68
% of area of necrosis
Vehicle n=3
BGJ398 n=4
Pemi n=4

Supplementary Figure 9: The effect of FGFR inhibition on the morphology of EC PDXs.

a) Representative images of vehicle-treated PDX tumours that were collected at day 23 (PDX59) and at day 22 (PDX60) when they reached maximum volume (900mm3), H/E examination showed solid growth of tumour with minimal necrosis or cysts (**top panels**) and marked central necrosis and cyst formation in EC PDX tumours treated with BGJ398 (PDX59) and Pemigatinib (PDX60) for 21 days and collected at survival endpoint which was 50 days (PDX59) and 70 days (PDX60) post-treatment completion (**Bottom panels**). b) Analyses showing the percentage area of necrosis of the tumour in the vehicle and FGFRi treated EC PDX models. PDX52, PDX59, PDX60 and PDX67 are PDX models of EC with FGFR2c splice isoform expression and PDX68 carries the C383R FGFR2 mutation. Bar indicates min and max values. PDX52, PDX59, and PDX68 were treated with BGJ398/infigratinib and PDX60 and PDX67 were treated with Pemigatinib. Scale bar indicates 100µm. DG, differentiated gland; CY cystic area; N, Necrosis

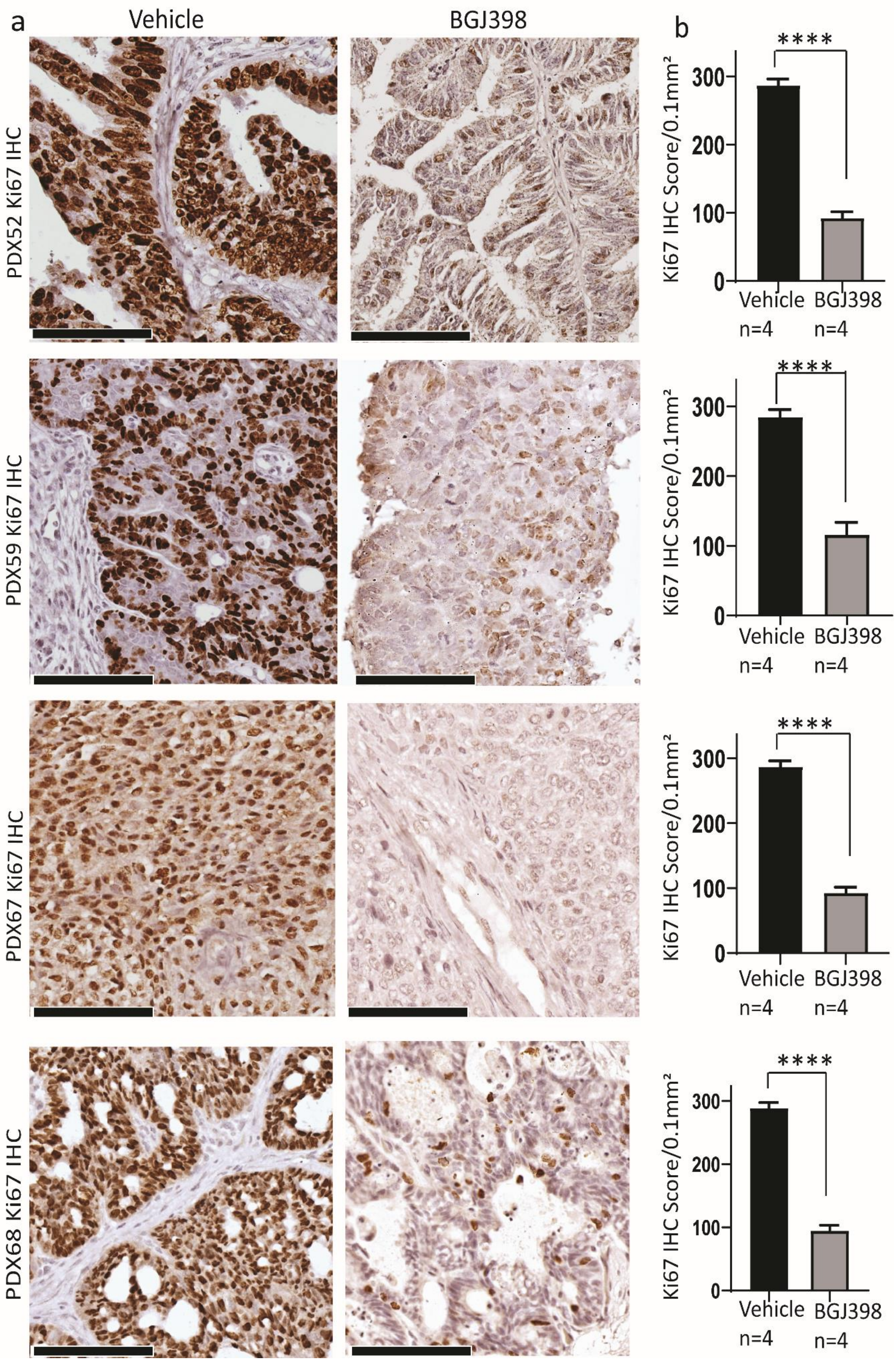
a
Vehicle
BGJ398
b
PDX52 Ki67 IHC
PDX59 Ki67 IHC
PDX67 Ki67 IHC
PDX68 Ki67 IHC
Ki67 IHC Score/0.1mm²
0
100
200
300
****
Vehicle
n=4
BGJ398
n=4

Supplementary Figure 10: Significant reduction of Ki67 expression following BGJ398 treatment in four EC PDX models.

a) Representative micrography images of Ki67 expression on tumours from four independent PDX models treated for 7 days with either vehicle (left panel) or BGJ398 (right panel) b) Quantification of Ki67 expression in BGJ398 versus vehicle treated tumours for each indicated model. PDX68 carries mutationally activated FGFR2 (C383R) while PDX52, PDX59 and PDX67 express FGFR2c. Ki67 was quantified using automated digital image analyses (Qu Path) and the score was reported as average/0.1mm^2 area. Significant differences were assessed with a two-sided student's T-test (**** P <0.0001) and error bars indicate the standard error of the mean (SEM), Scale bar 50µm.
